# A structural model of the iRhom–ADAM17 sheddase complex reveals functional insights into its trafficking and activity

**DOI:** 10.1007/s00018-023-04783-y

**Published:** 2023-04-29

**Authors:** Selcan Kahveci-Türköz, Katharina Bläsius, Justyna Wozniak, Cindy Rinkens, Anke Seifert, Petr Kasparek, Henrike Ohm, Shixin Oltzen, Martin Nieszporek, Nicole Schwarz, Aaron Babendreyer, Christian Preisinger, Radislav Sedlacek, Andreas Ludwig, Stefan Düsterhöft

**Affiliations:** 1 Institute of Molecular Pharmacology, Medical Faculty, RWTH Aachen University, Wendlingweg 2, 52074 Aachen, Germany; 2 Czech Centre for Phenogenomics, Institute of Molecular Genetics of the Czech Academy of Sciences, Prague, Czech Republic; 3 Institute of Molecular and Cellular Anatomy, Medical Faculty, RWTH Aachen University, Aachen, Germany; 4 Proteomics Facility, IZKF, RWTH Aachen University, Aachen, Germany

**Keywords:** iRhom, ADAM17, Ectodomain shedding, TNF signalling, EGFR ligand release iRhom–ADAM17 complex structure, iRhom homology domain

## Abstract

Several membrane-anchored signal mediators such as cytokines (e.g. TNFα) and growth factors are proteolytically shed from the cell surface by the metalloproteinase ADAM17, which, thus, has an essential role in inflammatory and developmental processes. The membrane proteins iRhom1 and iRhom2 are instrumental for the transport of ADAM17 to the cell surface and its regulation. However, the structure–function determinants of the iRhom–ADAM17 complex are poorly understood. We used AI-based modelling to gain insights into the structure–function relationship of this complex. We identified different regions in the iRhom homology domain (IRHD) that are differentially responsible for iRhom functions. We have supported the validity of the predicted structure–function determinants with several in vitro, ex vivo and in vivo approaches and demonstrated the regulatory role of the IRHD for iRhom–ADAM17 complex cohesion and forward trafficking. Overall, we provide mechanistic insights into the iRhom–ADAM17-mediated shedding event, which is at the centre of several important cytokine and growth factor pathways.

**Supplementary Information:**

The online version contains supplementary material available at 10.1007/s00018-023-04783-y.

## Introduction

Dysregulation of signalling is a hallmark of many pathologies including chronic inflammation and cancer. Therefore, the release of mediators such as cytokines and growth factors must be tightly controlled. An essential mechanism to generate signals is the proteolytic release (shedding) of ectodomains from membrane-bound mediator precursors on the cell surface. The proteases responsible for this are called sheddases. Many physiological relevant shedding events are facilitated by the transmembrane protease ADAM17 (A disintegrin and metalloproteinase 17).

ADAM17 is instrumental in the inflammatory response by releasing TNFα [1, 2] and its receptors as well as the interleukin 6 (IL6) receptor [3–5]. ADAM17 activity plays also an essential role in several epidermal growth factor receptor (EGFR) signalling pathways by shedding EGFR ligands including amphiregulin (AREG) or transforming growth factor (TGFα) [6, 7]. Absence of ADAM17 activity in mice results in developmental defects and lethality [7], while dysregulation of ADAM17 activity is implicated in pathologies such as chronic inflammation and cancer progression [4, 5, 8, 9]. Recently, ADAM17s diverse role in virus cell entry was discovered. ADAM17 can cleave the SARS-CoV-1/2 (severe acute respiratory syndrome coronavirus 1/2) receptor ACE2 and the SARS-CoV2 spike, which is an important requisite for efficient infections [10–14]. Furthermore, ADAM17 was identified as the entry factor of pestiviruses in cattle [15, 16]. Therefore, it is crucial to understand the regulation of ADAM17 in order to develop therapeutic approaches against ADAM17-dependent pathologies.

**Fig. 1:**
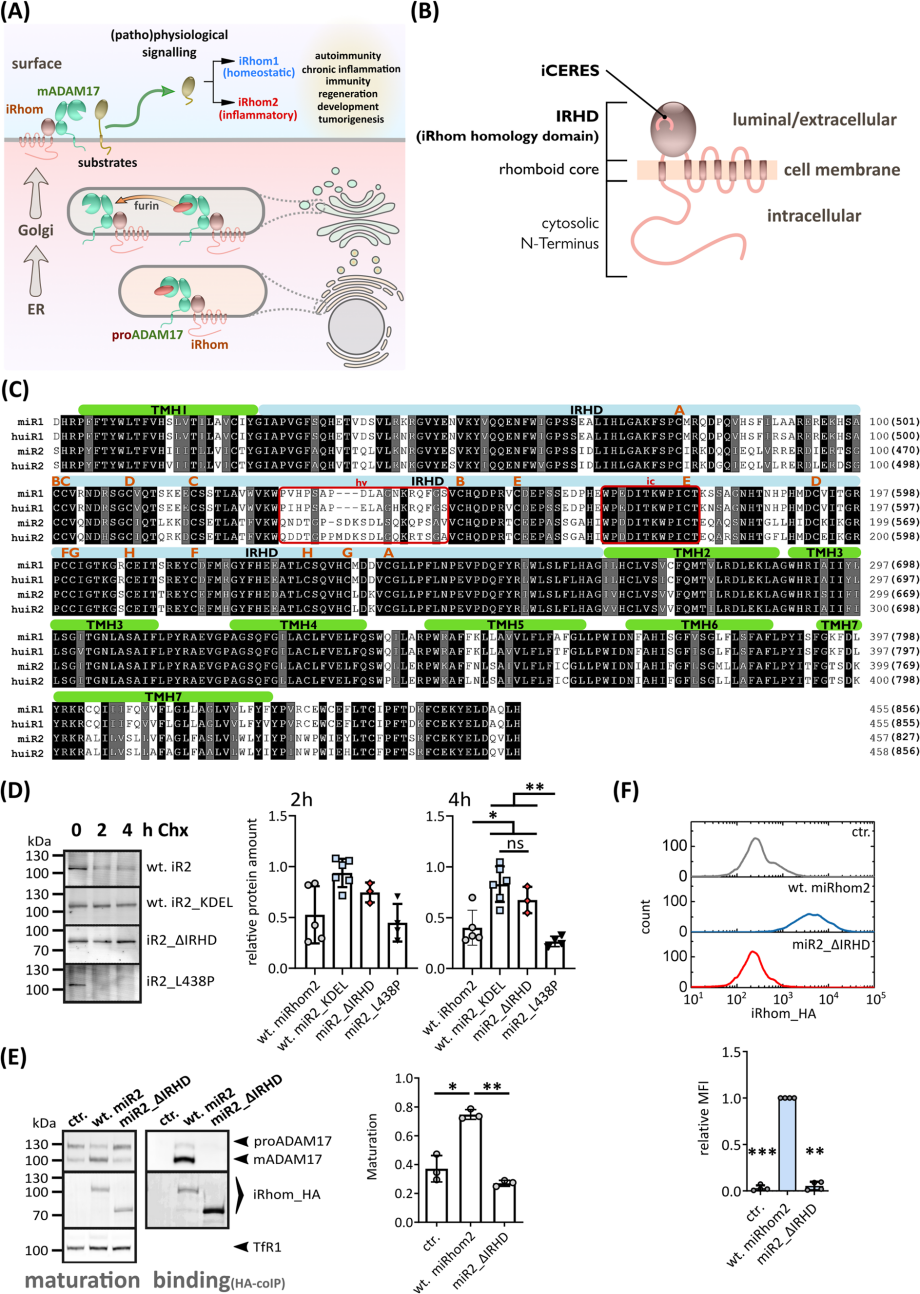
The IRHD is needed for ADAM17 binding, forward trafficking and maturation. **A** Role of iRhoms in the regulation of ADAM17: iRhoms bind immature proADAM17 in the ER and transport it to the Golgi, where maturation of ADAM17 takes place: A inhibitory prodomain (red) is proteolytically removed by furin-like proteases. Subsequently, mature ADAM17 can reach the cell surface and shed its substrates, facilitating a variety of different signalling pathways, such as the TNFα and EGFR signalling pathways. **B** Overview of the general iRhom topology. Structurally, iRhoms consist of the membrane-immersed rhomboid core of seven transmembrane helices (TMH), which is characteristic for rhomboids, and a cytosolic N-terminal tail. Between TMH1 and TMH2, the iRhom homology domain (IRHD) is located. **C** Sequence alignment of the IRHD (light blue) and rhomboid core (green) of both murine and human iRhom1 and iRhom2. Paired cysteine residues predicted (by the AlphaFold 2 model) to form disulphide bonds are indicated by letters. Variable sequence (hv) and conserved iCERES (ic) in IRHD are indicated by red boxes. **D** A cycloheximide-based pulse-chase experiment was performed to analyse the half-life of the indicated murine iRhom variants. *n* > 3. Cells were lysed at the indicated time points after initiation of treatment with cycloheximide (CHX) and subsequently analysed for iRhom2 by immunoblot. For each construct, immunoblot signals were quantified and calculated relative to the respective control without CHX at time 0 h, which was set to 1. **E** Immunoblot of samples from HEK293 cells stably expressing the indicated murine iRhom constructs or GFP (ctr.) as negative control were used. HEK293 cells have endogenous iRhoms and, therefore, exhibit a basal level of ADAM17 maturation. To analyse ADAM17 maturation, glycosylated proteins were enriched with concanavalin A beads. The maturation level of ADAM17 can be detected by the presence of mature ADAM17 (mADAM17) with lower molecular weight than immature proADAM17. The transferrin receptor (TfR1) served as an input control. ADAM17 maturation was assessed by densitometric measurements and calculation of the ratio between mADAM17 and total ADAM17 (derived by the sum of mADAM17 and imADAM17). *n* = 3. To analyse the binding between ADAM17 and iRhom constructs, coIPs were performed using the iRhom constructs (with HA tag) as bait. The quantitative analysis of ADAM17 binding can be found in Fig. S1A. *n* = 3. **F** Cell surface localisation of indicated constructs was measured by flow cytometry. For quantification, the geometric mean of the specific fluorescence signal was determined and normalised to the wt. *n* = 4

ADAM17, as a transmembrane protein, is not transported out of the ER after folding on its own. For many transmembrane proteins, it is still poorly understood how their ER exit is regulated. In the case of ADAM17, its immature proform forms a complex with iRhoms, inactive members of the rhomboid protease family, to initiate forward trafficking through the secretory pathway (Fig. 1A) [17–20]. In the Golgi, immature ADAM17 undergoes maturation and is then further transported to the cell surface (Fig. 1A). At the surface, the iRhom–ADAM17 complex persists and iRhoms are further involved in the regulation of ADAM17 activity and contribute to the stability of the complex [21–24].

Two iRhoms are present in mammals, namely iRhom1 (RHBDF1) and iRhom2 (RHBDF2). While iRhom1 is expressed in nearly all cells, iRhom2 seems to be particularly important in immune cells and under inflammatory conditions [17–20, 25–27]. The structural determinants for the cohesion of the iRhom–ADAM17 complex and how iRhoms facilitate the transport of ADAM17 are not yet known in detail. Recently, we found that a region within the iRhom homology domain (IRHD) of iRhom2, which we named iCERES (iRhom Conserved ER to Golgi Export Sequence), appears to be involved in its trafficking (Fig. 1B)[28]. Whether this function is preserved in iRhom1 has not been known so far.

Here, we present insights into the structure–function relationship of IRHD and the iRhom–ADAM17 complex. With the help of models generated with deep-learning-based structure predictions, we have analysed the iRhom–ADAM17 complex using different in vitro, ex vivo and in vivo approaches. We provide first evidence that the IRHD has a novel fold consisting of three substructures with distinct functions in ADAM17 regulation: an exposed conserved loop critical for forward transport efficiency, an exposed hypervariable loop and a highly structured subunit important for iRhom–ADAM17 interaction. Overall, our data point to distinct determinants in the IRHD for ADAM17 trafficking and binding that are relevant for the development of specific inhibitory approaches against ADAM17 activity.

## Results

### The IRHD is crucial for the iRhom–ADAM17 interaction

To date, the function of the IRHD, which is located between TMH1 and TMH2 of iRhoms, is not completely understood (Fig. 1B, C). It has previously been suggested that the IRHD may be involved in the iRhom–ADAM17 interaction [23, 24, 28]. However, complete deletions of the IRHD were used without testing whether it affects the stability of the remaining iRhom parts. To ensure that the rhomboid core can fold correctly it is important that there are enough amino acid residues to bridge the gap between TMH1 and TMH2 as it is the case with all members of the rhomboid family [29]. Therefore, we introduced a flexible linker (24 residues) in iRhom2 replacing the IRHD (miR2_ΔIRHD). We investigated the expression of miR2_ΔIRHD in HEK293 cells and tested its stability by performing cycloheximide-based pulse-chase experiments. The level of the full-length wt iRhom2 is significantly reduced after 4 h as it undergoes its normal turnover (Fig. 1D). In contrast, miR2-ΔIRHD is stable for hours, comparable to a wt iRhom2 construct that is C-terminally fused to the KDEL sequence, an ER retention signal, and thus trapped in the ER (Fig. 1D). Moreover, miR2_ΔIRHD is significantly more stable than an iRhom2 variant with a mutation that causes misfolding of the protein (Fig. 1D), as we have previously reported [28]. Hence, the exchange of the IRHD with a flexible linker does not cause protein instability.

As shown before, additionally expressed wt iRhom2 in HEK293 cells (with endogenous iRhoms present) binds ADAM17 (Fig. 1E, Fig. S1A) and promotes additional ADAM17 maturation compared to control cells (Fig. 1E) [28]. In contrast, while miR2_ΔIRHD is stable and still has a previously described ADAM17-binding interface within its TMH1 [24, 30], its interaction with ADAM17 is significantly impaired and thus miR2_ΔIRHD cannot promote ADAM17 forward trafficking and maturation (Fig. 1E). Moreover, miR2_ΔIRHD is not transported to the cell surface (Fig. 1F).

Overall, the IRHD is an important component for the cohesion of the iRhom2–ADAM17 complex and for its forward trafficking. However, it is not yet completely understood how the IRHD is involved in these functions.

### Deep-learning AI-based prediction provides structural insights into iRhoms

**Fig. 2:**
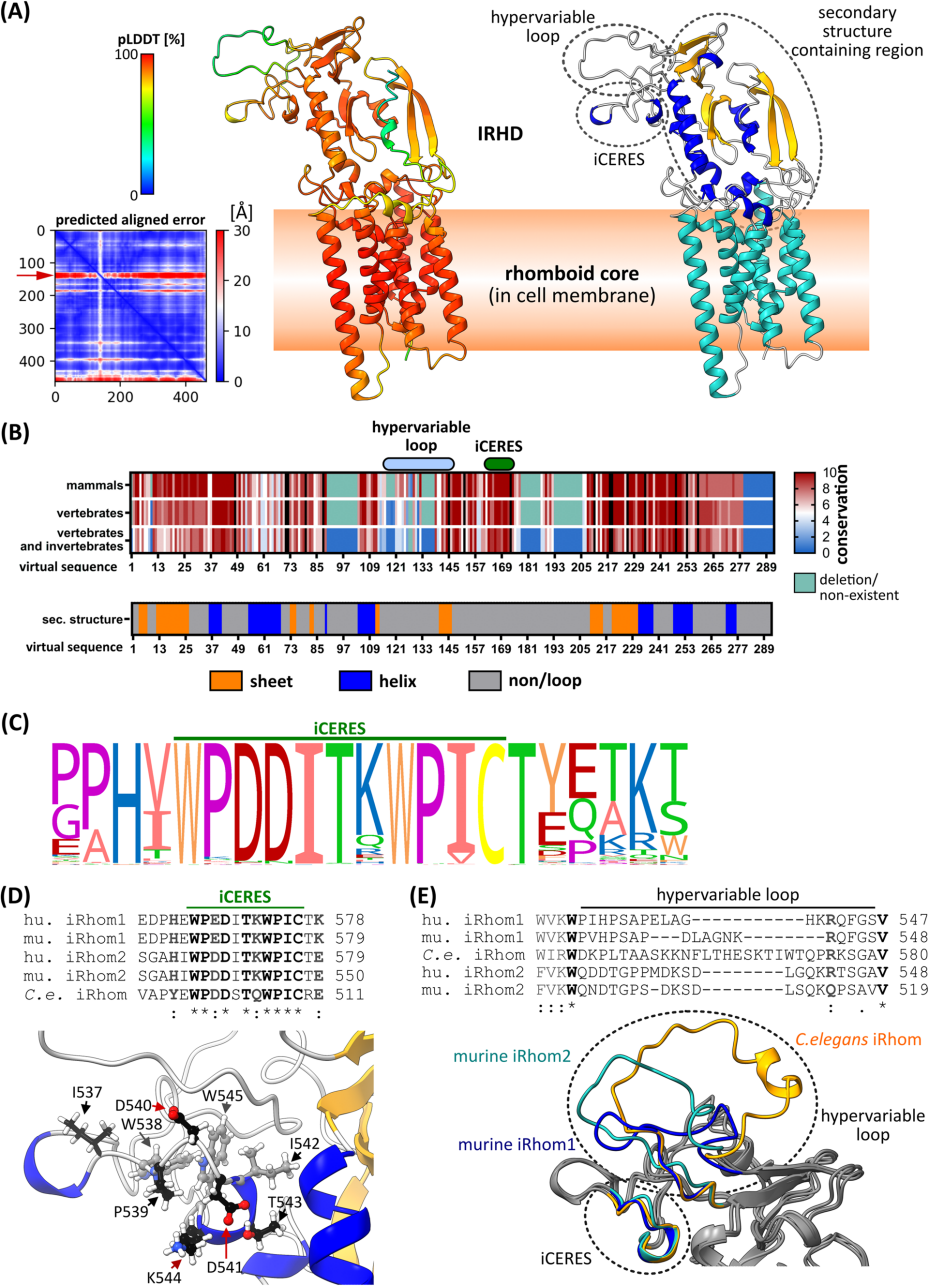
Ab initio structure prediction of iRhom IRHD and rhomboid core. **A** Ab initio structural modelling of iRhom2 without the cytosolic N-terminus using the deep learning algorithm AlphaFold 2 without homology templates. MMseq2 was used as the multiple sequence alignment. The structure model is predicted to be highly structured as indicated by the overall high pLDDT (predicted local distance difference test in [%]) score. The predicted align error (PAE) supports this: the individual amino acid residues are largely fixed in their position relative to each other and therefore have a low spatial uncertainty. The structure of iRhom2 IRHD and rhomboid core is depicted as cartoon representation with colouring corresponding to pLDDT score as well as corresponding to secondary structure (blue = helix, yellow = sheet, grey = loop). The IRHD can be divided in three parts: a highly structured part, the iCERES loop and a flexible, hypervariable loop. The flexibility of the hypervariable loop is indicated by a low pLDDT score and high PAE (red arrow). **B** Multiple sequence alignment of the IRHD of iRhom1 and iRhom2 from 30 different species as well as a corresponding secondary structure prediction. Conservation is demonstrated as heat map. Black lines in the alignment represent all cysteine residues—these are highly conserved. **C** We developed the algorithm CONYAR to retrieve all available amino acid sequences of a gene from UniProtKB and compare them to identify highly conserved regions within the amino acid sequences of that gene. In the case of iRhom2 (query: RHBDF2), sequences from 381 species were extracted. iCERES was identified by CONYAR as a highly conserved region and its conservation is shown as WebLogo representation. **D** Sequence alignment of iCERES from indicated species and structural representation of iCERES of murine iRhom2. **E** Sequence alignment of hypervariable loop from indicated species. Structural comparison of hypervariable loop and iCERES of indicated iRhoms. All structural models were generated with AlphaFold 2 as described in **A**

To gain further insights into the functions of the IRHD a structure is needed. However, no experimentally solved structure is available, nor have structural homologues been identified. Therefore, we used the deep-leaning AI AlphaFold 2 [31] to generate an ab initio structural model of the iRhom2 IRHD together with the rhomboid core (Fig. 2A). The prediction had a high degree of confidence for the rhomboid core and most parts of the IRHD, as shown by a high pLDDT (predicted Local Distance Difference Test in [%]) and a low PAE (Predicted Aligned Error) (Fig. 2A), as well as a favourable distribution of torsion angles (Fig. S1B). Furthermore, an independently derived in silico secondary structure prediction (Fig. 2B) is consistent with the modelled IRHD structure (Fig. 2A). Both predictions show that the IRHD of iRhom2 consists of a region with a high content of secondary structures, as well as two loops. One highly conserved loop includes iCERES, a region we recently described in iRhom2 [28], while the other loop is a hypervariable region indicated by its low pLDDT and high PAE (Fig. 2A, B). The IRHD structure and a differential multiple sequence alignment show that additional variable loop regions occur in iRhoms from invertebrates (Fig. 1C; Fig. 2A, B).

Since AlphaFold 2 and similar prediction methods such as TrRosetta [32] and RosettaFold [33] are based on the evolutionary conservation and do not recognise or take into account protein topologies, especially for membrane proteins, we used known properties of iRhom2 and (membrane-anchored) proteins in general as benchmarks to evaluate the accuracy of the structure prediction generated by AlphaFold 2: 1.] All seven transmembrane helices forming the hydrophobic rhomboid core lie in one plane, as would be expected since they should be immersed in the cell membrane (Fig. 2A; Fig. S1D). 2.] The surface of the predicted IRHD structure is primarily hydrophilic, consistent with its topology (Fig. S1D). 3.] All 16 cysteine residues of the IRHD are paired allowing the formation of eight disulphide bonds, as would be expected from a luminal/extracellular domain (Fig. 1C). Importantly, a model generated with RosettaFold [33] shows a very similar overall structure (Fig. S1C).

The AlphaFold 2 structure model shows also a high accuracy when we compare the modelled rhomboid core of iRhom2 with the experimentally determined structure of the rhomboid core of the *E. coli* rhomboid protease GlpG (Fig. S1E; Fig. S6), with an average error of 1.104 Å calculated using the root-mean-square deviation (RMSD) from the correct atomic positions (Fig. S6).

In addition, we have attempted to identify structural homologues of the IRHD in the Protein Data Bank (rcsb.org) [34] and in the AlphaFold Protein Structure Database [35] using Foldseek [36] and the Dali server [37]. The modelled structure of the iRhom2 IRHD appears to be a unique fold for the iRhom subfamily.

In summary, the structure of the rhomboid core and IRHD modelled ab initio has structural properties that are consistent with experimentally derived features. In addition, the modelled IRHD structure shows three substructures, that could be involved in iRhom functions: an iCERES-containing loop, a hypervariable loop and a large secondary structures-containing region.

### Structural model of the IRHD provides insights into iCERES as a general forward trafficking motif of the iRhom subfamily

The exposed iCERES-containing loop within the IRHD is formed by the disulfide bond C527–C548 and a hydrophobic core consisting of W538, I542 and W545 in murine iRhom2 (Fig. 2A, C, D; Fig. S1C). This explains why our previous mutation experiments in iRhom2 (W538S and W545S) caused the disruption of iCERES-dependent trafficking of the iRhom2–ADAM17 complex and, hence, ADAM17 maturation [28]. These mutations most likely impair the formation of the hydrophobic core and, thus, the functions of iCERES.

**Fig. 3:**
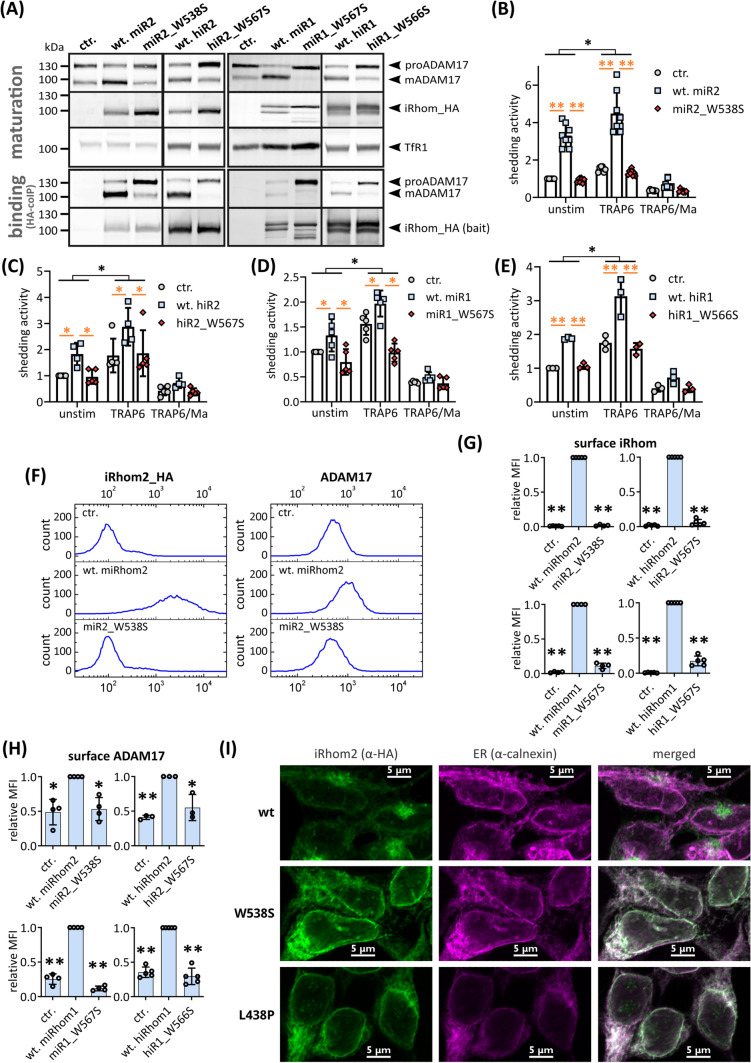
iCERES loop is a general motif in iRhoms crucial for ER-to-Golgi transport. HEK293 cells stably expressing the indicated iRhom constructs or GFP as negative control (ctr.) were used for the described experiments. The transferrin receptor (TfR1) served as input control. **A** Immunoblots to analyse the influence of the indicated iRhom constructs. Maturation was assessed as described in Fig. 1E. Quantification can be found in Fig. S2A. *n* > 4. To analyse the binding between ADAM17 and iRhom constructs, coIPs were performed by using the iRhom constructs (with HA tag) as bait. Quantitative analysis of ADAM17 binding can be found in Fig. S2B. *n* > 4. **B-E** ADAM17-mediated shedding activity was assessed by performing an alkaline phosphatase (AP) assay in HEK293 cells stably expressing the indicated iRhom construct: **B** murine iRhom2 (miR2), **C** human iRhom2 (hiR2), **D** murine iRhom1 (miR1), **E** human iRhom1 (hiR1). The ADAM17 substrate IL-1R_II_ tagged with AP was used. Since ADAM17 activity can be upregulated by stimulation of G-protein coupled receptors, HEK293 cells were stimulated with the PAR1 (protease activated receptor 1) agonist TRAP6. Additionally, the broad-spectrum metalloprotease inhibitor marimastat was used. Cells were incubated for 2 h under the indicated conditions. *n* > 3. **F–H** Cell surface localisation of indicated iRhom constructs and endogenous ADAM17 were measured by flow cytometry: **F** representative histogram of cells expressing murine iRhom2 variants. For quantification of ADAM17 surface localisation **G** and iRhom surface localisation **H**, the geometric mean of the fluorescence intensity was normalised to the corresponding wt iRhom sample. *n* > 3. **I** Confocal microscopy of cells expressing either wt iRhoms, the iCERES mutant miR2_W538S or the misfolded control mutant miR2_L438P. Antibody against HA tag was used to stain iRhom2 variants (green). Antibody against calnexin was used to stain ER (purple)

We also modelled the IRHD of murine iRhom1 and iRhom from *C. elegans.* iCERES shows high structural conservation between iRhom1 and iRhom2 as well as between iRhoms from different species, consistent with its sequence conservation (Fig. 2B–E). To test whether iCERES has also the same function in iRhom1, we also introduced iCERES inactivating mutations in iRhom1. Stable expression of the iCERES mutants murine iR2_W538S, human iR2_W567S, murine iR1_W567S and human iR1_W566S did not increase the amount of mature ADAM17 compared to the wt controls (Fig. 3A; Fig. S2A). Hence, disruption of iCERES in iRhom1 and iRhom2 blocks ADAM17 maturation. Importantly, iCERES mutants were still able to bind ADAM17, showing that the iCERES-containing loop is not involved in the interaction with ADAM17 (Fig. 3A; Fig. S2B).

Next, we analysed whether the iCERES mutations in murine and human iRhom1 influence the shedding of the known ADAM17 substrate IL1R_II_ [38]. In line with their effect on ADAM17 maturation, additional expression of wt iRhom2 and wt iRhom1 showed increased levels of constitutive IL1R_II_ shedding as well as induced IL1R_II_ shedding after stimulation with the PAR1 (protease activated receptor 1) agonist TRAP6 (Fig. 3B–E). In contrast, mutants with disrupted iCERES showed no increased ADAM17-mediated shedding compared to the respective wt iRhoms (Fig. 3B–E).

We also analysed the cell surface localisation of the iRhom mutants by flow cytometry in comparison to the respective wild type. While HA-tagged wt iRhom1 and iRhom2 could be detected on the cell surface (Fig. 3F, G; Fig. S2C-E), the surface localisation of the iCERES mutants is drastically reduced and corresponds closely to the negative control. As expected, surface ADAM17 is also increased when wt iRhom1 or iRhom2 are overexpressed, but not when iCERES mutants are present (Fig. 3F–H; Fig. S2C–E).

We additionally analysed the subcellular localisation of the iCERES mutant miR2_W538S by fluorescence microscopy. While miR2_W538S is localised only in the ER, wt iRhom2 is predominantly localised outside the ER in the Golgi (Fig. 3I; Fig. S2F). This is consistent with previous reports that ADAM17 and iRhom are mainly localised in intracellular compartments and only a subfraction is actual present on the cell surface [21, 28, 39–41]. As an additional control, we used an iRhom2 mutant (miR2_L438P), which, as we have previously described, causes the IRHD to misfold, resulting in ER retention and rapid ER-associated degradation [28]. Consistent with this, miR2_L438P was only found in the ER (Fig. 3I).

### iCERES is crucial for ADAM17-mediated cell functions

We used CRISPR/Cas9 to generate a knock-in mouse carrying the iCERES disrupting point mutation W538S in iRhom2 (Fig. S2G) as described before [28]. We isolated BMDMs (bone marrow-derived macrophages) from these mice. As in most immune cells, iRhom2 is predominantly expressed, whereas iRhom1 is absent [17]. Consistent with our in vitro experiments, the maturation of ADAM17 is impaired in BMDMs^WS/WS^ compared to BMDMs with wt iRhom2 (BMDMs^+/+^) (Fig. S2G). Therefore, the release of TNFα but not IL6 is significantly reduced in BMDMs^WS/WS^ after stimulation with the proinflammatory stimulus LPS compared to BMDMs^+/+^ (Fig. S2G). To further test the pathophysiological significance of iCERES, we performed a phagocytosis assay. We have recently reported that the absence of ADAM17 activity either through pharmacological inhibition or ADAM17 knock-out increases the phagocytosis rate of immune cells [42]. We tested BMDMs^WS/WS^ for their ability to phagocytose *E. coli*. In line with our previous results, BMDMs^WS/WS^ with abolished ADAM17 maturation also showed an increased phagocytosis rate compared to wt BMDMs (Fig. S2H).

Overall, iCERES is a shared and structurally conserved motif across the iRhom family that is essential for the forward trafficking of the iRhom–ADAM17 complex from the ER to the Golgi and thus for the maturation, surface localisation and shedding activity of ADAM17. iCERES is not only relevant for the regulation and activity of ADAM17 by promoting its forward trafficking in vitro, but also in (patho-)physiological settings.

### iCERES mutations differentially influence the trafficking efficiency of iRhoms

**Fig. 4:**
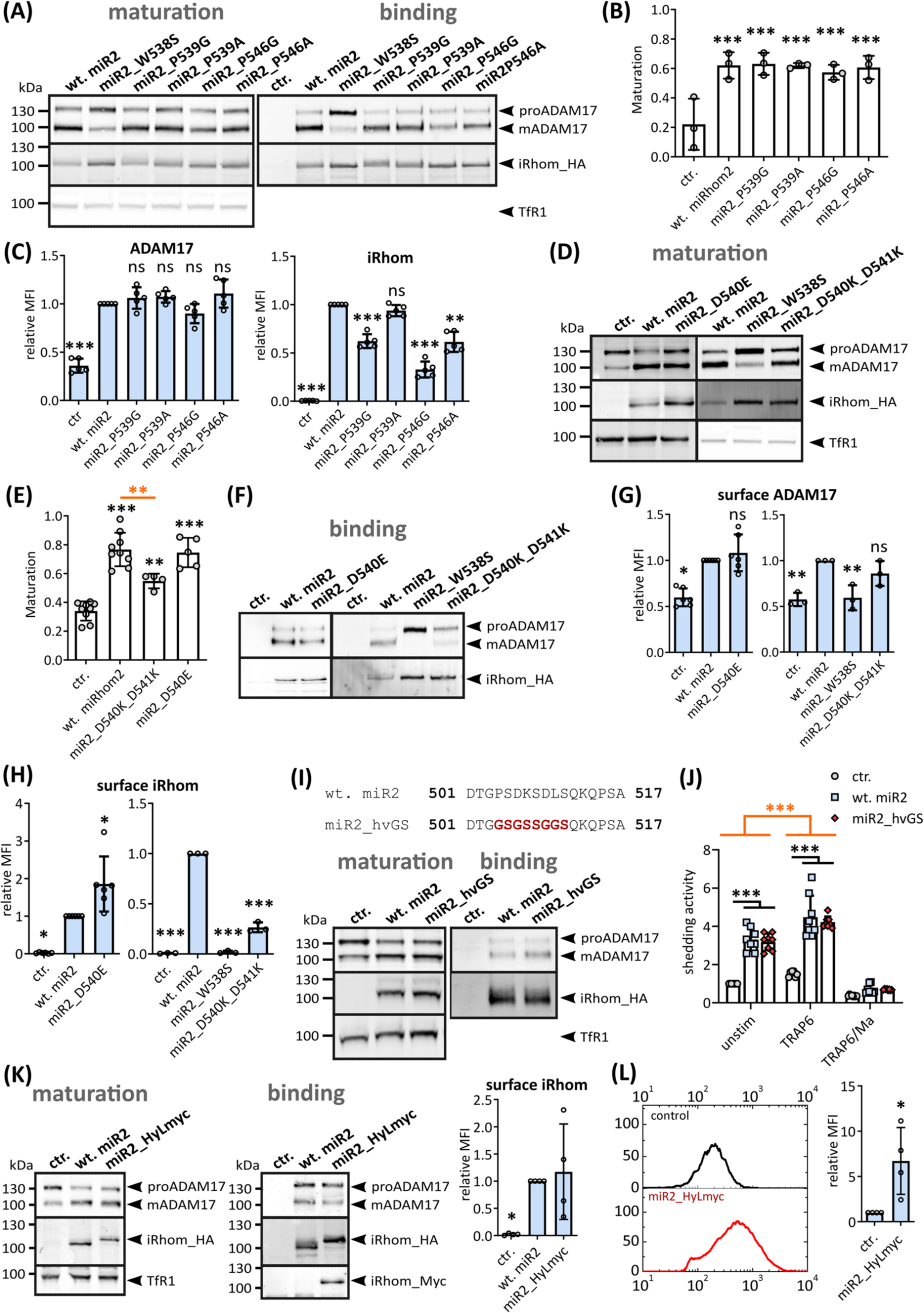
The iCERES loop and the hypervariable loop have distinct effects on the iRhom functions. HEK293 cells stably expressing the indicated murine iRhom constructs or GFP as negative control (ctr.) were used for the described experiments. The transferrin receptor (TfR1) served as input control. **A** Immunoblots to analyse the influence of the indicated iRhom P-mutants on ADAM17 maturation and binding. To analyse binding between ADAM17 and iRhom constructs, coIPs were performed using the iRhom constructs (with HA tag) as bait. The quantitative analysis of ADAM17 binding can be found in Fig. S4A. *n* = 3. **B** Maturation from **A** was assessed as described in Fig. 1E. *n* > 4. **C** Cell surface localisation of indicated iRhom P-mutants and endogenous ADAM17 were measured by flow cytometry. For quantification the geometric mean of the fluorescence intensity was normalised to the corresponding wt iRhom sample. *n* = 5. **D–E** Immunoblots to analyse the influence of the indicated iRhom D-mutants on ADAM17 binding. Quantification of maturation **E** was performed as described in Fig. 1E. *n* > 4.** F** Immunoblots to analyse the influence of the indicated iRhom D-mutants on ADAM17 binding (HA-coIP). The quantitative analysis of ADAM17 binding can be found in Fig. S4B. *n* > 3. **G–H** Cell surface localisation of indicated iRhom D-mutants **G** and endogenous ADAM17 (H) were measured by flow cytometry. For quantification, the geometric mean of the fluorescence intensity was normalised to the corresponding wt iRhom sample. *n* = 5. **I** Immunoblots to analyse the influence of miR2_hvGS with flexible GS-linker instead of hypervariable region on ADAM17 maturation and binding. *n* = 3. **J** Influence of miR2_hvGS on ADAM17-mediated shedding was analysed by performing an alkaline phosphatase (AP) assay. Cells expressing GFP were used as negative controls (ctr.). The ADAM17 substrate IL-1R_II_ tagged with AP was used. ADAM17 activity was additionally stimulated by TRAP6 or inhibited by marimastat. Cells were incubated for 2 h under indicated treatment. *n* = 8. **K** Immunoblots to analyse the influence of miR2_HyLmyc with 3 × myc tag instead of hypervariable region on the maturation and binding of ADAM17. *n* = 3. Additionally, cell surface localisation of miR2_HyLmyc was measured by flow cytometry. For quantification the geometric mean of the fluorescence intensity was normalised to the corresponding wt iRhom sample. *n* = 4. **K** Surface exposure of the 3 × myc tag of miR2_HyLmyc was measured by flow cytometry. For quantification the geometric mean of the fluorescence intensity was normalised to the staining negative control (ctr.). *n* = 4

We have demonstrated that mutations in the iCERES region neither cause protein instability nor affect binding to ADAM17 (Fig. 3A) [28]. Parts of iCERES may resemble a C-mannosylation motif. This post-translational modification has been shown to regulate ER exit [43–45]. However, we found no evidence that iCERES functions are C-mannosylation-dependent (Fig. S3A, B). iCERES may represent a regulatory and/or interaction site for a yet unidentified factor. To further test the importance of the local structure of the iCERES-containing loop, we targeted the two proline residues, as proline residues are known to be important for short loop formations [46]. We mutated the proline residues P539 and P546 in iRhom2 to either glycine or alanine (P-mutations). Neither binding to ADAM17, ADAM17 maturation nor ADAM17 surface localisation appeared to be affected by the P-mutations compared to wt iRhom2 (Fig. 4A, B, C; Fig. S4A). However, the iRhom2 P-mutations themselves show reduced surface localisation, with the exception of P539A (Fig. 4C). These results show that the residual forward trafficking of the P-mutants is sufficient to allow the maximum possible ADAM17 forward trafficking.

While proline, tryptophan and isoleucine residues within the iCERES loop appear to be necessary for its structure, the residues D540, D541 and K544 with their exposed side chain may be more directly involved in facilitating the forward trafficking of iRhom (Fig. 2C; Fig. S1C). Interestingly, the first aspartate residue (D540) in iRhom2 is replaced by a glutamate residue in the iRhom1 sequence (Fig. 2C). To analyse the effect of this difference, we generated the murine iRhom2_D540E mutant. Neither ADAM17 binding nor ADAM17 maturation was affected by this mutation compared to wt iRhom2 (Fig. 4D, E, F; Fig. S4B). However, the D540E mutation resulted in a significant increase in surface iRhom2 compared to wt iRhom2, indicating more efficient forward transport (Fig. 4H). However, surface ADAM17 was not affected (Fig. 4G). This is consistent with our earlier observation that the maximal amount of surface ADAM17 has already been reached. In contrast, the introduction of combined drastic charge-reversing mutations D540K and D541K into murine iRhom2 (miR2_D540K_D541K) significantly reduces the efficiency of forward trafficking and thus the surface localisation of iRhom2 (Fig. 4H) compared to wt iRhom2 without affecting ADAM17 binding (Fig. 4F; Fig. S4B). However, more iRhom2_D540K_D541K is detectable on the surface than iRhom2_W538S (Fig. 4H), which again is sufficient to promote increased ADAM17 maturation (Fig. 4D, E) and elevated ADAM17 levels on the surface, albeit to a lesser extent than wt iRhom2 (Fig. 4D, E, G).

Overall, the different positions of the iCERES-containing loop differentially control the forward trafficking efficiency of iRhoms.

### Identification of an exposed hypervariable loop in the IRHD supports structure prediction

In contrast to the conserved iCERES-containing loop, the second exposed loop is predicted as unstructured/disordered in the modelled structures of murine iRhom1, murine iRhom2 and *C. elegans* iRhom and shows high evolutionary sequence variability (Fig. 1C; Fig. 2A, B, D, E). To investigate whether the hypervariable loop affects iRhom functions and shedding efficiency, we replaced it with a flexible linker in iRhom2 (miR2_hvGS) (Fig. 4I). miR2_hvGS showed the same ADAM17-binding capacity and ADAM17 maturation efficiency as wt iRhom2 (Fig. 4I). Moreover, miR2_hvGS expression resulted in the same increased constitutive and TRAP-6-induced shedding of IL1R_II_ and of AREG as wt iRhom2 compared to control cells (Fig. 4J; Fig. S4C). In addition, we expressed miR2_hvGS in MEFs (mouse embryonic fibroblasts) derived from mice deficient for iRhom1 and iRhom2 [19]. miR2_hvGS was able to rescue iRhom knock-out by promoting ADAM17 maturation and ADAM17-mediated shedding of TNFα, comparable to wt iRhom2 (Fig. S4D, E). In contrast, the iCERES disrupting mutant miR2_W538S failed to rescue the knock-out phenotype (Fig. S4D, E).

Since iRhoms seem to tolerate mutations of the hypervariable loop, we additionally inserted a 3xmyc tag (additional 34 residues) (Fig. S4F) to analyse whether this loop is really exposed as shown by the modelled structure of the IRHD (Fig. 2E). Again, these changes in the hypervariable loop do not alter iRhom2 forward trafficking, ADAM17 binding or the facilitation of ADAM17 maturation compared to wt iRhom2 (Fig. 4K). Furthermore, we were able to detect the myc tag by flow cytometric measurements, proving that this loop is indeed exposed as demonstrated in the IRHD structure (Fig. 4L).

### Structural modelling of the iRhom–ADAM17 complex reveals determinants of interaction

**Fig. 5:**
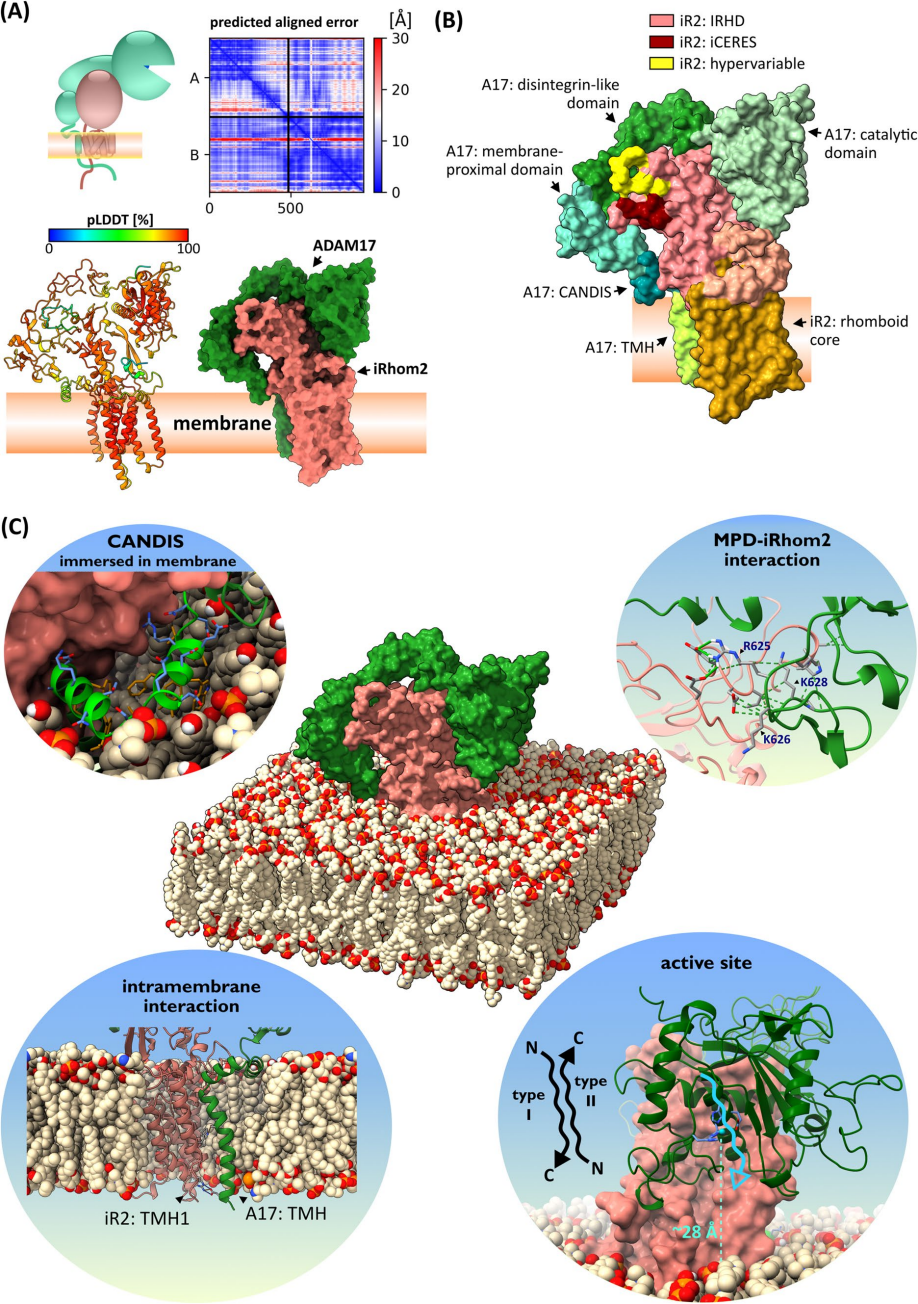
Ab initio structure prediction of iRhom–ADAM17 complex. **A** Ab initio structural modelling of murine iRhom2 together with murine ADAM17 without the cytosolic tails using the deep learning algorithm AlphaFold Multimer without homology templates. The structure of the complex is predicted to be highly structured, as indicated by the overall high pLDDT score [%]. This is confirmed by the low intramolecular PAE. The high abundance of low intermolecular PAE indicates multiple binding sites between ADAM17 and iRhom2, particularly between the TMH1 and ADAM17 TMH and between the IRHD and ADAM17 ectodomain. The structure of the complex is shown as a cartoon representation with colouring according to the pLDDT score and as a surface representation (green = ADAM17, salmon = iRhom2). While only the top-ranked structure prediction model is shown, the other lower-ranked models look very similar (Fig. S7). The pdb files of all ranked models of the AlphaFold multimer structure predictions for the murine iRhom2–mature ADAM17 complex and the murine iRhom–proADAM17 complex, respectively, can be found in the supplementary data. **B** Surface representation of the modelled iRhom2–ADAM17 complex with colour coding of the substructures as indicated. **C** Surface representation of the modelled iRhom2–ADAM17 complex embedded in a modelled membrane (4 × POPC and 1 × Chol). The following features of the predicted iRhom2–ADAM17 complex are highlighted: (1) immersion of CANDIS into the membrane, (2) putative binding interface (hydrogen bonds and Van der Waals contacts) between MPD of ADAM17 and IRHD of iRhom2, (3) TMH of ADAM17 and rhomboid core of iRhom2, (4) catalytic domain of ADM17 showing necessary positioning of substrates in the active site of ADAM17 (HExxHxxGxxH coordinates Zn^2+^) to be shed

We have demonstrated that neither the iCERES loop nor the hypervariable loop in the IRHD is involved in ADAM17 binding. To identify potential binding interfaces between ADAM17 and iRhoms we generate an ab initio structural model of murine ADAM17 together with iRhom2 with AlphaFold Multimer [47]. Strikingly, both proteins are predicted as a tight complex with high accuracy, as indicated by the overall high pLDDT score and the low intermolecular PAE, especially for the contacts between both proteins (Fig. 5A, B; Fig. S5A; Fig. 7A–D, Table S1). We additionally modelled the iRhom–ADAM17 complex with murine iRhom1 and murine ADAM17, with human iRhom1 and human ADAM17, with human iRhom2 and human ADAM17, and with the evolutionarily distant ADAM17 and iRhom from Caenorhabditis elegans (Fig. S8). All these combinations resulted in very similar iRhom–ADAM17 complex structure predictions (Fig. S9). Importantly, modelling a complex of murine iRhom2 with murine ADAM10 with AlphaFold Multimer did not predict an interaction, consistent with previous findings that ADAM17 is the only member of the ADAM family that interacts with iRhoms [17] (Fig. S5B). In addition, the modelled structure of the iRhom–ADAM17 complex shows high accuracy compared to already experimentally solved structures of domains of ADAM17 and its close structural relative ADAM10 [48–51] (Fig. S6). In the modelled structure iCERES and the hypervariable loop are still exposed and predicted to be not involved in ADAM17 binding (Fig. 5B), which is consistent with our experimental findings.

Overall, the modelled complex structure predicts that the ectodomain of ADAM17 binds in a C-shape around the IRHD. This C-shape has already been shown for other ADAMs and has also been proposed for ADAM17 (Fig. 5A, B) [50–53]. We also added a membrane environment to the complex (Fig. 5C) by using the PPM 2.0 Web Server and the CHARMM-GUI Bilayer Builder [54–56]. Noteworthy, the previously experimentally determined interaction of ADAM17 TMH with TMH1 of iRhom2 [24, 30] was also correctly predicted (Fig. 5A, B, C). AlphaFold Multimer predicts several additional interaction interfaces linking the IRHD of iRhom2 with the membrane-proximal domain (MPD), the disintegrin-like domain, the catalytic domain and the prodomain of ADAM17 (Table S1). This prediction of the IRHD as an additional determinant of iRhom–ADAM17 complex cohesion (Fig. 5A, B; Fig. S5A; Fig. S7A–D, Table S1) is consistent with our findings that iRhom2 lacking the IRHD is insufficient to efficiently bind ADAM17 (Fig. 1E).

Interestingly, according to our structural model, the catalytic domain and its active site are about 28 Å displaced from the cell membrane (Fig. 5C). Since most cleavage sites of ADAM17 substrates are proximal to the membrane, the spatial hindrance prevents the shedding process from occurring without conformational changes. This is consistent with the observation that ADAM17 must be activated for shedding to occur, resulting in a change in the ADAM17 structure that brings the catalytic domain closer to the membrane [48, 53, 57–63]. Consistent with this, we have previously shown that for type I transmembrane substrates, non-induced ADAM17-mediated shedding increases when the cleavage site is moved further away from the membrane [61]. Type II transmembrane substrates are even more spatially hindered, as they must enter the active site of ADAM17 in the same N-to-C-terminal direction (Fig. 5C). We tested whether moving the cleavage site C-terminally away from the membrane would also increase the non-induced shedding of type II transmembrane proteins (Fig. S5C). And indeed, the further the cleavage site is moved away from the membrane, the higher the shedding efficiency (Fig. S5C), which supports the structural model of the iRhom–ADAM17 complex (Fig. 5C).

## Discussion

**Fig. 6:**
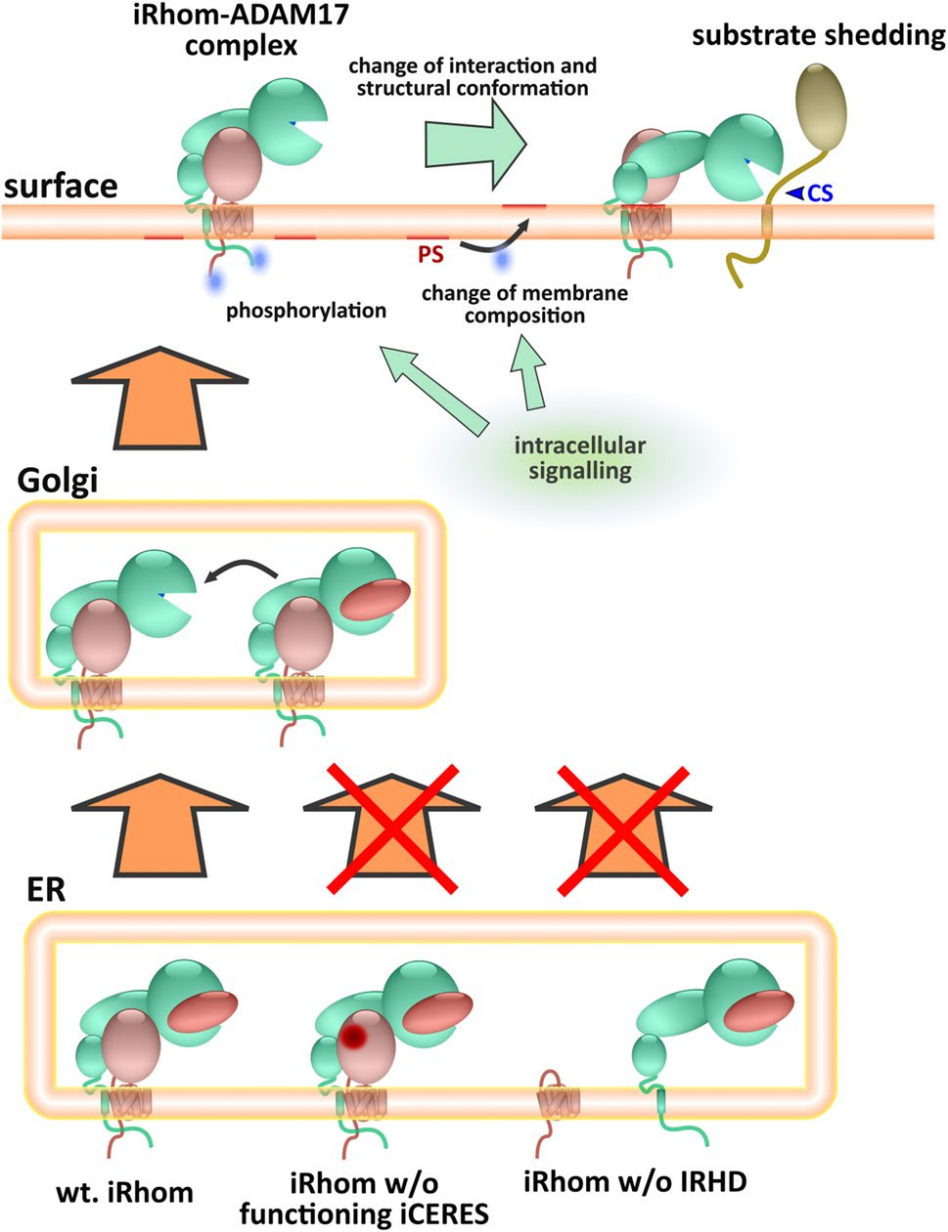
IRHD is essential for iRhom–ADAM17 complex cohesion, forward trafficking and shedding activity. Without IRHD, no stable complex of iRhom and immature ADAM17 (with prodomain) can be formed. Without a functioning iCERES loop in the IRHD, no efficient transport of the iRhom–ADAM17 complex to the Golgi can take place. In the Golgi, the maturation of ADAM17 occurs (removal of the prodomain). At the cell surface, the shedding event is caused by intracellular signals, changes in membrane composition and conformational changes

Understanding the regulation of the iRhom–ADAM17 complex is important due to its central role in several cytokine and growth factor pathway such as TNFα and EGFR signalling. Hence, modulating its function has therapeutic potential in several pathologies including chronic inflammation and cancer. However, needed insights into the structure–function relationship of this complex were missing. Our structural models revealed three distinct regions within the so far poorly characterised iRhom homology domain: a highly structured part, a hypervariable flexible loop and a conserved loop. The conserved loop contains a region called iCERES (iRhom Conserved ER to Golgi Export Sequence)[28]. Here, we demonstrated that iCERES represents a structurally exposed loop that is highly conserved at the sequence and structural level in iRhom2 and iRhom1 in both vertebrates and invertebrates. Our results show that this motif is critical for ER-to-Golgi transport of the iRhom–ADAM17 complex and thus for ADAM17 maturation and shedding activity (Fig. 6). Mutation of iRhom2 iCERES in mice abolished iRhom2-dependent ADAM17-mediated shedding comparable to complete iRhom2 knock-out mice [17, 18, 28, 42, 64], directly affecting ADAM17-dependent processes such as phagocytosis [42]. Mutations of iCERES positions not involved in the hydrophobic core of the conserved loop can differentially decrease or increase the efficiency of iRhom forward trafficking. Importantly, these mutations do not affect ADAM17 binding, consistent with the structural model of the whole iRhom–ADAM17 complex. Overall, the iCERES loop has regulatory properties and we can only speculate that it may represent an interaction interface of a yet unknown factor required for the transport of the iRhom–ADAM17 complex to the Golgi. While the cytosolic motifs in transmembrane proteins that are important for forward trafficking are well established, luminal motifs are less well known. Further studies are needed to understand the underlying mechanism.

The AlphaFold structural model also predicted a hypervariable loop that is exposed even when iRhom is in complex with ADAM17. We were able to demonstrate experimentally that this loop is indeed exposed at the protein surface, but is not involved in ADAM17 binding. Since it has been described that iRhom1 and iRhom2 are differentially involved in the substrate selectivity of ADAM17 [65, 66], sequence differences as in the hypervariable loop are primary targets to understand variations in shedding efficiency with distinct substrates. However, we have shown that the hypervariable loop has no effect on shedding efficiency of AREG, which was reported to be a iRhom2-dependent substrate [66]. Nevertheless, since this loop differs even between iRhom1 and iRhom2 in one species, it represents an excellent target for an iRhom1- or iRhom2-specific antibody and perhaps even an inhibitory bispecific antibody against the entire iRhom2–ADAM17 or iRhom1–ADAM17 complex when combined with known ADAM17-inhibitory antibodies [67, 68].

While individual domains of ADAM17 have been solved experimentally [48–50], a complete experimentally derived structure is still missing, especially in complex with iRhoms, but is urgently needed to address mechanistic questions. We have used AlphaFold to gain some initial insights into the structure of the complex. Importantly, the predicted structure is consistent with the substructures already solved and with experimental results, including the involvement of IRHD in the ADAM17 interaction (Fig. 5C; Fig. 6). Furthermore, this model is also in line with previous results including the interaction interface between the ADAM17 TMH and TMH1 of iRhoms [24, 30]. We have previously identified a motif in the stalk region of ADAM17, which we have termed CANDIS (Conserved ADAM 17 Dynamic Interaction Sequence), representing an amphipathic helix immersed in the cell membrane [62, 63]. Again, the modelled structure of the iRhom–ADAM17 complex is consistent with our experimental results: CANDIS is indeed modelled as an amphipathic helix in the correct orientation and position in which it would be immersed in the lipid bilayer (Fig. 5C).

However, a limitation of the AlphaFold is that it only produces one preferred structural conformation. Therefore, the predicted iRhom2–ADAM17 complex structure must be considered in the molecular context. Originally, it was assumed that iRhom is mainly important for the transport of ADAM17 into the Golgi and to the surface [17–19, 27, 64]. Mature ADAM17 on the surface has to be activated to perform the shedding process [8, 57, 59, 69]. Here, different aspects of the regulation of ADAM17 activation and substrate recognition were identified such as binding of tissue inhibitor of metalloproteinases (TIMP)3 [70], binding of α5β1-integrin [71, 72], the redox environment and protein disulphide isomerase activation [48, 63, 73–75] and phosphatidylserine exposure [60, 76]. These results support the hypothetical model that the activation of shedding requires a flexible structure of the ADAM17 ectodomain, allowing a conformational change that permits the catalytic domain to reach the substrate cleavage sites, whereas a rigid structure would prevent shedding [61, 62, 76]. However, these results were largely obtained either with isolated domains and substructures or with ADAM17 alone, neglecting a possible influence of iRhoms. Recently, the direct involvement of iRhoms in the shedding process was revealed. Here, it was shown that iRhom phosphorylation appears to reduce the affinity between iRhom and ADAM17 [23, 24], presumably leading to greater structural flexibility of ADAM17. In addition, it was shown that iRhom2 may be involved in the substrate selectivity of ADAM17 [65, 66]. Therefore, iRhoms also seem to be an inhibitory factor for the activity of ADAM17.

With respect to these earlier findings, we hypothesise that the modelled structure (Fig. 5A–C) represents the non-activated sheddase complex, in which the ADAM17 structure is rigid and locked in place by iRhom. The predicted position of the catalytic domain and hence its active site are unable to reach the membrane-proximal cleavage sites of its substrates (Fig. 5C; Fig. 6) [8, 53, 60, 77–78]. This is consistent with our experimental results showing that moving cleavage sites of type I transmembrane substrates [61] and of type II transmembrane substrates increases non-induced shedding efficiency, proving that the active site is accessible but spatially hindered under non-induced conditions.

One of the predicted interaction interfaces between the IRHD and ADAM17 involves the RKGK (R625 to K628) sequence of ADAM17 (Fig. 5C, Fig. S7A–D, Table S1). These residues are part of a regulatory motif in the MPD that binds exposed phosphatidylserines on the membrane and induces a conformational shift that is a prerequisite for the shedding process [60, 62, 76]. Hence, the modelled complex structure (Fig. 5A–C) suggests that iRhoms lock the ADAM17 conformation and block the R625–K626–K628 motif. This is indeed also in line with recent reports, in which it was shown that ADAM17 is tightly bound to iRhoms on the cell surface, but that a weakening of this interaction upon iRhom phosphorylation is necessary to initiate the shedding process [23, 24]. However, it is important to note that the identified interaction sites, including the ADAM17-RKGK motif, are based on the relaxed iRhom2–ADAM17 complex structure model (Fig. S7A–D, Table S1) and that molecular dynamics simulations should be performed in future studies to further characterise the interaction sites.

Overall, we propose the following model (Fig. 6): In the ER, iRhoms interact with the immature proADAM17. The IRHD is required for efficient cohesion of the iRhom–ADAM17 complex, and iCERES in particular is required to allow transport to the Golgi. In the Golgi, proADAM17 is matured by furin-like proteases. Under non-stimulated conditions on the cell surface, ADAM17 is tightly bound to iRhom and therefore cannot engage with its substrates. Intracellular signals cause phosphorylation of the cytosolic N-terminus of iRhom. This process leads to a weakening of the cohesion of the iRhom–ADAM17 complex and promotes flexibility of the ADAM17 ectodomain. Intracellular signals also result in a transient phosphatidylserine exposure on the cell surface. This enables a conformational change in ADAM17 promoted by phosphatidylserine-dependent membrane binding, allowing the active site of ADAM17 to reach the membrane-proximal cleavage sites of its substrates.

It must be emphasised that this sequence of events is only speculative, but it is consistent with our results as well as with all data published so far. Although our results already allow first insights into the shedding event, further experimental investigations of the structure of iRhom and ADAM17 should provide more detailed insights into this process.

Chronic inflammatory diseases including rheumatoid arthritis, psoriasis and inflammatory bowel disease are among the leading causes of death today, and millions of people suffer from their symptoms [79–81]. So far, several biologics against TNFα such as infliximab are used as drugs to reduce chronic inflammations. However, in mouse models, a therapeutic approach to directly block the formation of soluble TNFα proved to be more effective [82].Yet, all attempts to find inhibitors with high specificity against the ADAM17 active site have failed. By expanding the insight into the mechanisms of iRhom and ADAM17 regulation, our results provide additional targets for potential therapeutic strategies. Disruption of iCERES functions could be one such potential target, although further insights into the mechanism are required. A more immediate target is the hypervariable loop as an antibody epitope. A bispecific antibody against the ectodomain of ADAM17 and the hypervariable loop would selectively bind only iRhom1 or iRhom2, potentially locking the complex in its inactive conformation. This is of particular interest as iRhom2 is predominantly involved in proinflammatory processes, whereas iRhom1 is mainly involved in homeostatic processes. [17–19, 27, 83]. Furthermore, strategies targeting iRhom functions to modulate ADAM17 activity and cell surface localisation may have beneficial implications for the prevention and treatment of viral diseases in humans and cattle due to the role of ADAM17 in SARS-CoV1/2 and pestivirus infections, respectively [10–16]. Finally, beyond the proinflammatory signalling and virus infections, dysregulated ADAM17 is involved in cancer progression through proteolytic release of EGF receptor ligands [9, 67, 84, 85]. Hence, blocking ADAM17 by targeting iRhoms is also a promising treatment strategy against cancer progression.

## Materials and methods

### Alignments and secondary structure prediction

Multiple sequence alignments and secondary structure prediction were generated utilising Clustal Omega [86], PRALINE [87] and Jpred4 [88]. The following sequences were used (UniProtKB entries): Q96CC6, Q6PJF5, Q6PIX5, Q80WQ6, A7YWH9, E1BLR4, L9KXR9, L9L0J9, F6VNW2, F6ZHX0, G1U9B8, G1T7M2, W5PLR9, W5PAM2, G7NGY1, F6ZPC8, A0A2K5TX09, G7Q013, H2NPI3, E1C4R0, E1BVU5, A0A094K3Y4, I3KUU1, I3KPE4, I3IYX4, Q6GMF8, H3BCD1, H3B6F2, A0A087XXE2, A0A0F8BD72, F7CCK3, A0JPA1, A0A151P699, A0A151N300, Q76NQ1, F1KU35, A0A0N4U485, Q9U2S3, B7QLY4, E2ASS8, A0A0P5YH19, A0A0V1CNV0, A0A0K8TBK6 and A0A0V0RFC9.

### Algorithm for high-throughput identification of conserved sequences

Complementing the traditional manual sequence alignment method to identify conserved sequence regions, an automated method for high-throughput comparison of as many sequences as possible was also used. For this purpose, the specially developed Python script CONYAR (Conserved You Are) was employed (https://git.rwth-aachen.de/ababendreyer/CONYAR). CONYAR retrieves all hits of a search query with the gene name of the desired protein from the UniProt Knowledgebase via the REST-API https://rest.uniprot.org/uniprotkb. The results are then filtered so that only the longest sequence with the highest annotation score is used for each species. In addition, all entries are removed that contain a sequence with a length less than 50% of the median of the sequences of all entries. Then, using Biopython [89], Clustal Omega [86, 90] is used to perform a multiple sequence alignment. Subsequently, the conserved regions with a minimum length of 3 amino acids and a threshold of 0.6 were identified using Biopython. To visualise the alignments and the conserved regions, the R package ggmsa [91] was used with the help of Rpy2.

### Structure prediction and modelling

Ab initio structures of iRhom and iRhom–ADAM17 complex (without cytosolic regions) were generated with AlphaFold 2, AlphaFold Multimer, RoseTTAFold and TrRosetta [31–33, 47, 92] utilising ColabFold [93]. Structural modelling using the deep learning algorithm AlphaFold 2 was done without homology templates. MMseq2 was used as the multiple sequence alignment. The structural modelling went through 12–24 iterations for higher accuracy. The respective top-ranked models were used. AlphaFold Multimer version 2 (v2) was used when not otherwise stated. For structural relaxation (five consecutive times) of side chains FoldX5 forcefield was used [94]. To model the iRhom–ADAM17 complex in a lipid bilayer consisting of the phosphatidylcholine 1-Palmitoyl-2-oleoylphosphatidylcholine (POPC) and cholesterol (Chol) the PPM 2.0 Web Server and CHARMM-GUI Bilayer builder were used [54–56]. ChimeraX was used to analyse and illustration of protein structures [95]. For calculating coulombic electrostatic potential ChimeraX was used. To compare/superimpose protein structures and calculate RMSD (root-mean-square deviation) ChimeraX matchmaker algorithm was used. ChimeraX [95] and InterProSurf [96] were used to analyse interaction interfaces (hydrogen bonds, Van der Waals contacts) in the predicted complex models.

### Cloning

The cloning procedures were performed as previously described [28]: NEBuilder HiFi DNA Assembly Master Mix (NEB, E2621L) was used to generate different plasmids with the desired inserts according to the manufacturer’s instructions. All iRhom constructs were cloned into the pMOWS backbone [97], with either puromycin resistance or zeocin resistance. Site-directed mutagenesis was performed using overlapping PCR [98].

### Cell culture

Culturing of HEK293 cells and MEFs, double deficient for iRhom1 and iRhom2 (MEF_dKO): in a humidified incubator at 37 °C, 5% CO_2_ in DMEM10%, which consists of DMEM high-glucose (Sigma-Aldrich) supplemented with 10% foetal calf serum (PanBiotech), 100 mg/l streptomycin (Sigma-Aldrich) and 60 mg/l penicillin (Sigma-Aldrich). Generating of stable cells was performed as described before [28]. MEFs were obtained from indicated mouse lines as described before [19]. BMDMs were freshly isolated from femur and tibiae of respective mouse lines as described previously [42]. HEK293 cells were purchased from German Collection of Microorganisms and Cell Cultures (GmbH DSMZ- No. ACC 305).

### Co-immunoprecipitation and enrichment of glycosylated proteins

For precipitation experiments, 8.0 × 10^6^ cells were lysed in 1 ml lysis buffer (50 mM Tris; 137 mM NaCl; 2 mM EDTA; 10 mM 1,10-Phenanthroline; pH7.5) supplemented with cOmplete™ protease inhibitor (Sigma; 11,697,498,001). Cell lysates were cleared by centrifugation at 16,000 × g, 15 min at 4 ℃. 450 µl cleared lysate was used for enrichment of glycosylated proteins with 30 µl Concanavalin A sepharose (Sigma; C9017) or co-immunoprecipitations (coIPs) with 10 µl anti-HA magnetic beads (ThermoFisher; 88,836). The respective lysates were incubated with beads for 90 min and subsequently washed 5 × with lysis buffer. Beads were cooked in 40 µl reducing loading buffer (3% (w/v) SDS, 16% glycerol, 8% 2-mercaptoethanol, 0.01% (w/v) bromophenol blue, 0.1 M Tris HCl, pH 6.8) at 65 °C for 20 min.

### Western blotting

Samples were separated by SDS-PAGE and transferred onto polyvinylidene difluoride (PVDF) membranes (Millipore, Immobilon-FL). Blocking of membranes was done with 5% (w/v) non-fat dry milk in TBS (50 mM Tris, 150 mM NaCl, pH7.4) for 20 min at room temperature. Incubation with primary antibodies was done in 0.1% Tween-TBS and 1% (w/v) BSA overnight at 4 °C. After three times washing with 0.1% Tween-TBS the membrane was incubated with secondary antibody for 1 h at room temperature. After additional washing steps once with 0.1% Tween-TBS and three times with TBS, protein detection was done via the Odyssey 9120 imager system (LI-COR) and the ChemiDoc MP Imaging System (Bio-Rad). For quantification, the band intensities were measured with the software Image Studio Lite (LI-COR). The following primary antibodies were utilised: αADAM17 (1:1000; Abcam; ab39162), αHA (1:2000; Biolegend; 901502), αTransferrin-receptor (1:2000; Abcam; ab1086), αiRhom2 (1:2000; Sigma; SAB1304414). The following secondary antibodies with the indicated dilutions were used: DyLight-680-conjugated αmouse (1:20000; Thermo; 35519), DyLight-800-conjugated αrabbit (1:20000; Thermo; 35571), horse radish peroxidase (HRP)-conjugated goat αmouse and αrabbit (1:20000; Jackson ImmunoResearch Laboratories, Inc).

### Shedding activity: AP assay

ADAM17-mediated shedding activity was measured by an alkaline phosphatase (AP)-based assay as described before [28]. In brief, cells transfected with the indicated ADAM17 substrate were stimulated with 100 nM PMA (Sigma; P1585) or 30 µM TRAP6 (Bachem; H-2936), or not stimulated (treated with vehicle DMSO). The metalloprotease inhibitor marimastat (broad-spectrum, 10 µM) (Sigma; M2699) or TAPI1 (inhibitor preferentially against ADAM17, 10 µM) (Sigma; SML0739) was used as a control. Cells were incubated for 120 min at 37 °C. Proteolytic activity was assessed by measuring AP activity in the supernatant and lysates (lysis buffer: 50 mM Tris; 137 mM NaCl; 2 mM EDTA; 10 mM 1,10-phenanthroline; pH7.5). Addition of p-nitrophenyl phosphate (PNPP) solution (Thermo; 37620) allowed AP activity to be measured continuously at 405 nm using the FLUOstar Optima (BMG LABTECH). The slope (change in absorbance at 405 nm per minute) was calculated to evaluate the AP activity. ADAM17 activity was calculated as PNPP substrate turnover (AP activity) in the supernatant relative to total turnover in the supernatant plus cell lysate. The following ADAM17 substrates cloned in pCDNA3.1 were used: AP-IL1R_II_, AP-TNFα, AP-AREG.

### Flow cytometric analysis

The assay buffer used was PBS with 0.2% BSA and all steps of the staining process were performed at 4 °C or on ice. 2 × 10^5^ cells of interest were incubated with the primary antibody for 1 h. The cells were then washed twice with 400 μl assay buffer. Secondary antibody was added and cells were incubated in the dark for 45 min. After two more washing steps, the fluorescence signal was analysed by flow cytometry (LSRFortessa, BD Biosciences, Heidelberg, Germany) and analysed using FlowJo V10 software. The geometric mean of the fluorescence intensity was determined to identify the cell surface localisation. The following primary antibodies were used at the indicated dilutions: αADAM17 (1:100; R&D Systems; MAB 9301), αHA (1:500; Biolegend; 901,502), αmyc (1:200; abcam; ab32). The following secondary antibodies were used at the indicated dilutions: Allophycocyanin-conjugated αmouse (1:200; Jackson ImmunoResearch; 115–135-164).

### Immunocytochemistry

For immunofluorescence staining, cells stably expressing the construct of interest were seeded onto glass coverslips as follows. The glass coverslips were prepared by rinsing with ddH_2_O and 70% ethanol and sterilising. The sterile glass coverslips were incubated for 30 min at 37 °C with 1 ml of 1% collagen G solution (in PBS) in 12-well plates. After incubation, the glass coverslips were washed with PBS. 1 × 10^5^ cells were added to each well in DMEM10%. The cells were incubated for 2–3 days at 37 °C and 5% CO_2_ to 80% confluence. For fixation, the medium was removed and the cells were washed with PBS. 0.5 ml/well of 4% paraformaldehyde (PFA) was added and incubated for 20 min at room temperature. After removal of the 4% PFA solution, the cells were washed with PBS. For antibody staining, primary antibodies were diluted in 0.1% Triton-X100 PBS: mouse αHA (1:200; Biolegend; MMS-101P), rabbit αCalnexin (1:500; Abcam; ab22595), rabbit αGM130 (1:500; Cell Signaling Technology; 12,480). Fixed cells on coverslips were briefly washed twice with PBS and then immersed in 0.1% Triton-PBS. Cells were stained by incubation with 100 μl antibody solution for 1 h and washed by immersion in PBS. Cells were then incubated for 1 h in the dark with 100 µl secondary antibody solutions: goat αmouse Alexa 488 (1:300; MoleProbes; A21121), donkey αrabbit Alexa 647 (1:250; Thermo; A32795). Afterwards, the cells were washed in PBS. Glass coverslips were then immersed in ddH_2_O and placed on a drop of mounting medium (Shandon Immu-Mount; Thermo Scientific; 10,622,689). The samples were stored at 4 °C in the dark. Imaging was done on a Zeiss LSM710 Duo microscope equipped with an oil immersion objective (63x/1.40-N.A. DIC M27) and an AiryScan detector (Zeiss). Fluorescent signal was recorded with the Airyscan detector in “superresolution” mode. For Alexa 488 fluorescence an argon-ion laser was used at 488 nm and for Alexa 647 fluorescence a 633 nm HeNe laser was used. Gain and laser intensities were optimised for best image quality. Microscopy images were processed and analysed with Zeiss Fiji (ImageJ) [99].

### Cycloheximide-based pulse-chase experiment

2 × 10^6^ HEK293 cells expressing the indicated iRhom variant were seeded 24 h before the pulse. During the pulse, cells were treated with 10 µg/ml cycloheximide (CHX) for the indicated periods. Cells were harvested and recounted to adjust all samples to an equal cell number. Harvested cell samples were lysed and used for IP protein enrichment as previously described.

### BMDM isolation

BMDMs were isolated out of femur and tibia of hind limbs of 8–10-week-old mice as described before [28]. The muscle tissue was removed and the bones were stored in cold PBS throughout the isolation procedure. The femur and tibia were cut open at both ends and flushed with ice-cold RPMI1640_FPS (RPMI1640 supplemented with 10% foetal calf serum, 100 mg/l streptomycin and 60 mg/l penicillin) using a syringe (18-G needle). Cells were centrifuged at 300 g for 5 min at 4 °C and resuspended in culture medium (RPMI1640_FCS and 20% L929-conditioned medium) using a syringe (26-G needle). Cells were spread on two 15 cm cell culture dishes per mouse in 20 ml culture medium. After 72 h, 10 ml of culture medium was added and changed on day 6. On day 10, BMDMs were seeded in 12-well plates. During stimulation, BMDMs were cultured for 24 h in the absence of L929-conditioned medium. At the end of differentiation, BMDMs were mainly (more than 90%) F4/80- and CD11b positive, as determined by flow cytometry. For LPS stimulation, BMDMS were treated with 100 ng/ml LPS for 8 h.

### Shedding activity in BMDMs

5 × 10^5^ BMDMs/well were seeded 24 h before stimulation in 2 ml fully supplemented growth medium. The medium was then replaced (750 µl) and cells were stimulated for 24 h with 100 ng/ml LPS from *E. coli* strain 0127:B8 (Sigma-Aldrich; L4516). The release of murine IL6 and murine TNFα into the supernatant of stimulated BMDMs was analysed using commercial ELISA kits (R&D Systems, DuoSet) according to the manufacturer’s protocols. The analysis was performed with a FLUOstar OPTIMA (BMG-Labtech).

### Phagocytosis assay

Phagocytosis assay was performed as described earlier [42]. Briefly, for phagocytosis assays, 2 × 10^5^ BMDM/ml were seeded 24 h before infection. *E. coli* pHrodo-conjugates are not fluorescent at neutral pH (outside the cell) but become fluorescent at acidic pH (phagosomes). The uptake of the particles was analysed after 1 h. The cells were washed with PBS and harvested. The fluorescence signal of the control cells (without bacteria) was subtracted from the corresponding samples. If BMDMs were infected with *E. coli* GFP, the medium was replaced with antibiotic-free RPMI supplemented with 10% FCS. *E. coli* GFP bacteria were freshly grown in Lennox-L-Broth (LB) medium containing 0.2 mM isopropyl-β-D-1-thiogalactopyranoside (IPTG) to initiate GFP expression to early log growth, resuspended in the appropriate antibiotic-free medium supplemented with 10% FCS and used immediately. Infection was performed with a multiplicity of infection (MOI) of 25 or 50.

### Statistics

Statistic was done as described before [28]: all experiments were repeated at least three times as indicated in the figure legends. Quantitative data are shown as mean with standard deviation (SD). Statistics were conducted using the generalised mixed model analysis (PROC GLIMMIX, SAS 9.4, SAS Institute Inc., Cary, North Carolina, USA) and assumed to be from either normal, lognormal or beta distribution with the day of experiment as random to assess differences in the size of treatment effects across the results. Residual analysis and the Shapiro–Wilk test were used as diagnostics. In the case of heteroscedasticity (according to the covtest statement) the degrees of freedom were adjusted by the Kenward–Roger approximation. All *p* values were adjusted for multiple comparisons by the false discovery rate (FDR). *p* < 0.05 was considered significant with *p* * < 0.05, ** < 0.01, *** < 0.001.

## Supplementary Information

Below is the link to the electronic supplementary material.

Supplementary file1 (PDB 1415 KB)

Supplementary file2 (PDB 1169 KB)

Supplementary file3 (PDB 1169 KB)

Supplementary file4 (PDB 1169 KB)

Supplementary file5 (PDB 1169 KB)

Supplementary file6 (PDB 1169 KB)

Supplementary file7 (PDB 1415 KB)

Supplementary file8 (PDB 1415 KB)

Supplementary file9 (PDB 1415 KB)

Supplementary file10 (PDB 1415 KB)

Supplementary file11 (PDF 4632 KB)

Supplementary file12 (PDF 246 KB)

## Data Availability

The datasets generated during the current study are available from the corresponding author upon request.

## Abbreviations

ADAM: A disintegrin and metalloproteinases
AREG: Amphiregulin
coIP: Co-immunoprecipitation
EGFR: Epidermal growth factor receptor
iCERES: iRhom conserved ER to golgi export sequence
IL6: Interleukin 6
IRHD: iRhom homology domain
MPD: Membrane-proximal domain
PAE: Predicted aligned error
pLDDT: Predicted local distance difference test
TGFα: Transforming growth factor alpha
TNFα: Tumour necrosis factor alpha
