# Supplementary material for: A structural model of the iRhom–ADAM17 sheddase complex reveals functional insights into its trafficking and activity
Source: Cell Mol Life Sci. 2023 Apr 29;80(5):135. doi: 10.1007/s00018-023-04783-y (PMC10148629; doi:10.1007/s00018-023-04783-y)
Supplement: Supplementary file 11 — Supplementary file11 (PDF 4632 KB) [file 18_2023_4783_MOESM11_ESM.pdf]

Figure S1: (A) Quantification of coIPs (Fig. 1E). **(B)** Ramachandran plot of murine iRhom2 structure generated with AlphaFold 2 (Fig. 2A). **(C)** Comparison of the IRHD of murine iRhom2 generated with either AlphaFold 2 (rainbow colour) or RosettaFold (light blue). The IRHD structure generated with AlphaFold 2 is shown as a cartoon coloured according to the calculated RMSD of the structure comparison (good match/ RMSD = 0 Å: blue; poor match/ RMSD ≥ 10 Å: yellow). **(D)** Surface representation of modelled iRhom2 structure without cytosolic tail coloured according to electrostatic potential or hydrophobicity. **(E)** Comparison of iRhom2 without cytosolic tail generated with either AlphaFold 2 (rainbow colour) or TrRosetta (grey) with TMH1 in pink. **(F)** Comparison of modelled murine iRhom2 structure with experimentally solved structure of *E.coli* GlpG (pdb: 3zmh). Structures are depicted as cartoon representation. Superposition of GlpG rhomboid core (grey) and iRhom2 rhomboid core (rainbow coloured). In addition, the rhomboid core of iRhom2 is coloured according to the calculated RMSD of the structural comparison.

Fig. S1

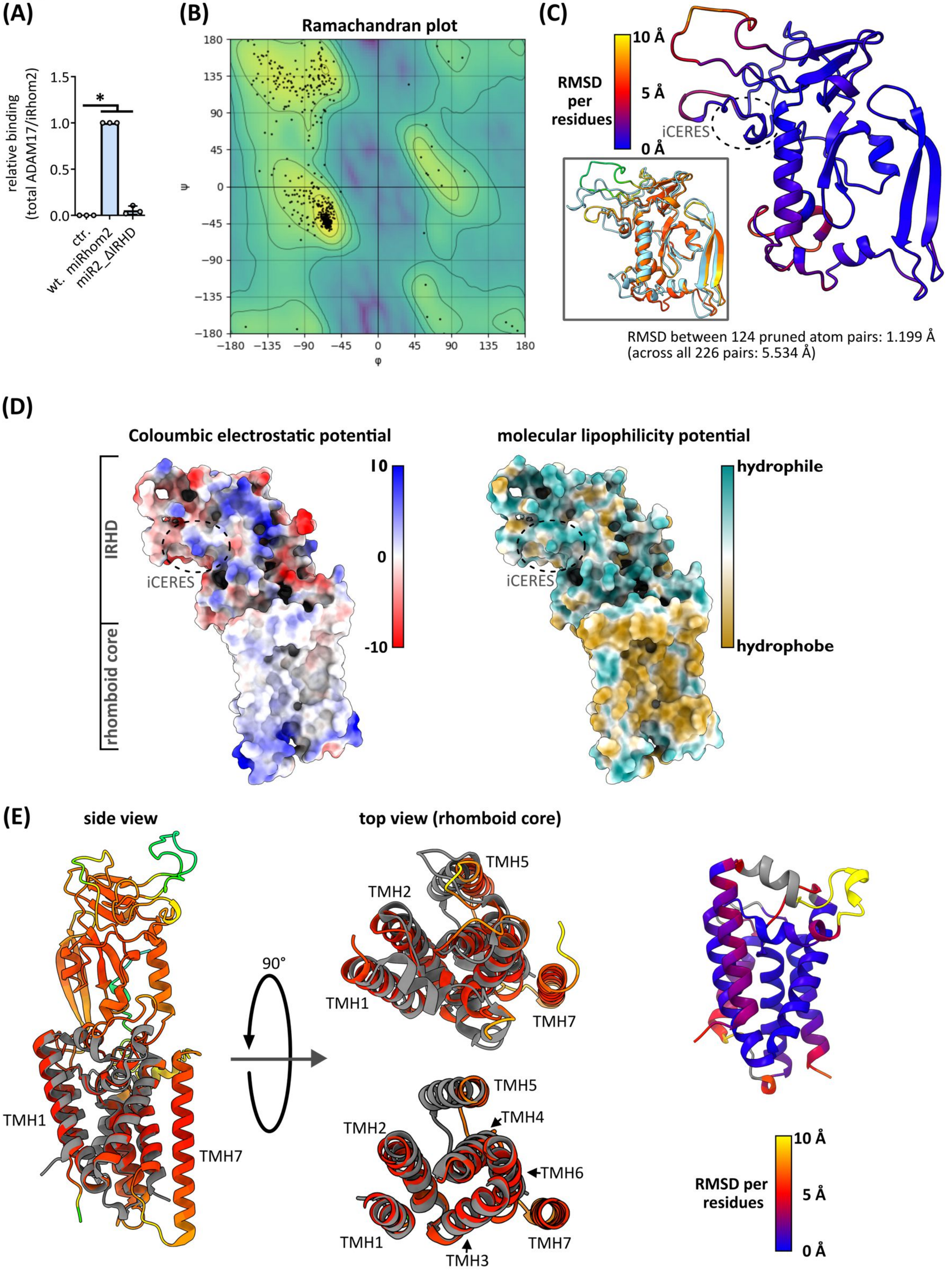

(A)
relative binding (total ADAM17/iRhom2)
1.5
1.0
0.5
0.0
*
ctr.
wt. miRhom2
miR2_ΔIRHD
(B)
Ramachandran plot
180
135
90
45
0
−45
−90
−135
−180
ψ
φ
(C)
10 Å
5 Å
0 Å
RMSD per residues
iCERES
RMSD between 124 pruned atom pairs: 1.199 Å
(across all 226 pairs: 5.534 Å)
(D)
Coloumbic electrostatic potential
molecular lipophilicity potential
IRHD
rhomboid core
iCERES
10
0
-10
hydrophile
hydrophobe
(E)
side view
top view (rhomboid core)
90°
TMH1
TMH2
TMH3
TMH4
TMH5
TMH6
TMH7
RMSD per residues
10 Å
5 Å
0 Å

Figure S2: (A) Quantification of effect of indicated iRhom variants on ADAM17 maturation (Fig. 3A). **(B)** Quantification of coIPs (Fig. 1A). **(C-E)** Representative flow cytometry histograms: surface localisation of indicated iRhom variants and their effects on surface ADAM17. **(F)** Confocal microscopy of cells expressing wt iRhoms. Antibody against HA tag was used to stain wt iRhom2 (green). Antibody against the Golgi-resident GM130 was used to stain Golgi (purple). **(G)** Wt mice and mice carrying the W538S (WS) mutation in iRhom2 were used to isolate BMDMs. Immunoblot demonstrates presence of wt iRhom2 and iRhom2 with W538S mutation in respective BMDMs (antibody against endogenous iRhom2). No mature ADAM17 was detected in BMDMs from mice homozygous for the W538S mutation. The transferrin receptor (TfR1) served as input control. BMDMs with W538S mutation show normal IL6 but impaired TNFα release 24 h after proinflammatory LPS (100 ng/ml) treatment. n = 5. **(H)** Phagocytosis assay with wt BMDMs and BMDMs from mice with W538S mutation in iRhom2. Representative histograms and quantification of phagocytosis assay are shown. *E.coli* with either pHrodo or GFP were used. For quantification the geometric mean of the fluorescence intensity was used. n >6.

Fig. S2

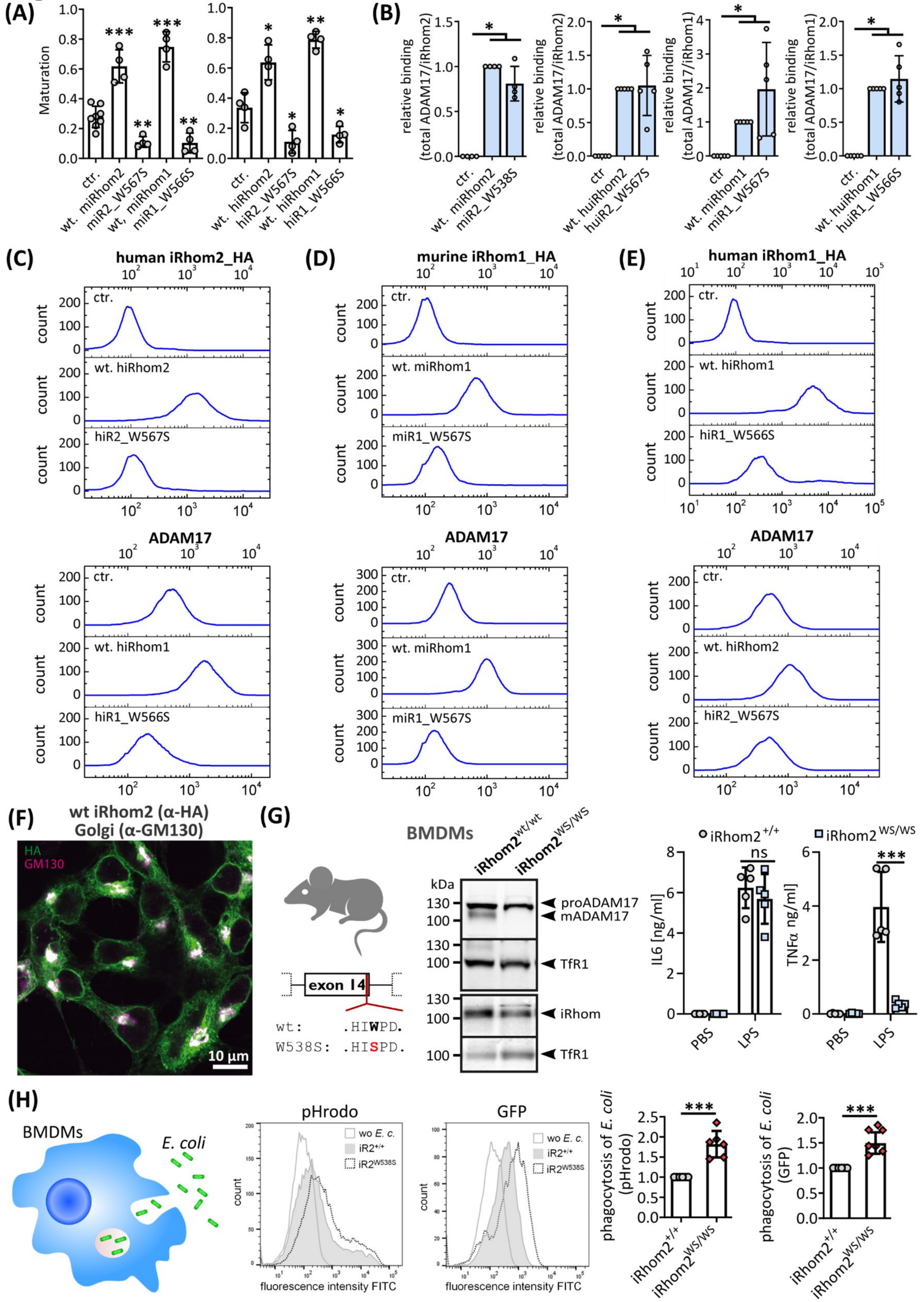

(A)
Maturation
ctr.
wt. miRhom2
miR2_W567S
wt. miRhom1
miR1_W566S
wt. hiRhom2
hiR2_W567S
wt. hiRhom1
hiR1_W566S
(B)
relative binding (total ADAM17/iRhom2)
relative binding (total ADAM17/iRhom1)
miR2_W538S
wt. huiRhom2
huiR2_W567S
miR1_W567S
wt. huiRhom1
huiR1_W566S
(C)
human iRhom2_HA
(D)
murine iRhom1_HA
(E)
human iRhom1_HA
ADAM17
count
(F)
wt iRhom2 (α-HA)
Golgi (α-GM130)
HA
GM130
10 µm
(G)
BMDMs
exon 14
wt: .HIWPD.
W538S: .HISPD.
iRhom2wt/wt
iRhom2WS/WS
kDa
proADAM17
mADAM17
TfR1
iRhom
iRhom2+/+
iRhom2WS/WS
IL6 [ng/ml]
TNFα ng/ml]
ns
PBS
LPS
(H)
BMDMs
E. coli
pHrodo
GFP
wo E. c.
iR2+/+
iR2W538S
fluorescence intensity FITC
phagocytosis of E. coli (pHrodo)
phagocytosis of E. coli (GFP)

Figure S3: Noteworthy, parts of iCERES resemble the C-mannosylation motif WXXC, which is a posttranslational modification of a tryptophan residue [43, 44] and has been shown to be a critical factor for ER exit. Recently, Shcherbakova, Preller [45] reported that C-mannosylation supports the stability of proteins in the ER. Thus, we investigated whether C-mannosylation could be involved in iCERES-dependent forward trafficking of the iRhom-ADAM17 complex by promoting protein stability. We screened wt iRhom2 for C-mannosylation by mass spectrometry but detected only non-mannosylated peptides. **(A)** Introduction of classical C-mannosylation motifs into murine iRhom2 variants with iCERES-disrupting single point mutations (W545S) did not rescue forward trafficking: HEK293 cells stably expressing the indicated murine iRhom2 mutants or GFP as negative control (ctr.) were used for the described experiments. The transferrin receptor (TfR1) served as input control. Immunoblots to analyse the influence of the indicated iRhom2 mutants on ADAM17 maturation and binding are shown. To analyse binding between ADAM17 and iRhom2 mutants, coIPs were performed using the iRhom mutants (with HA tag) as bait. n = 3. **(B)** These results are in line with our modelled IRHD structure, since both tryptophane residues in question are forming a hydrophobic core, which is not accessible for C2-mannosylation. In contrast, tryptophane residues in the thrombospondin type 1 repeats are exposed and hence accessible for C-mannosylation: Cartoon representation of IRHD (murine iRhom2; AlphaFold 2 prediction) and thrombospondin type 1 repeat (TSR1) of UNC5 (pdb: 4v2a). Tryptophane residues (putatively) involved in C-mannosylation are depicted in red.

Fig. S3

(A) known C-mannosylation motifs: WXXWXXW
WXXW
WXXC

wt. miRhom2	535	SSGAHIWPDDITKWPIC	548
miR2_W545S	535	SSGAHIWPDDITKSPIC	548
miR2_D541W_W545S	535	SSGAHIWPDWITKSPIC	548
miR2_S532W_A535W_W545S	535	WSGWHIWPDDITKSPIC	548
wt. miRhom2	501	DTGPSDKSDLSQKQPSA	517
miR2_D506W_D509W_W545S	501	DTGPSWKSWLSQKQPSA	517

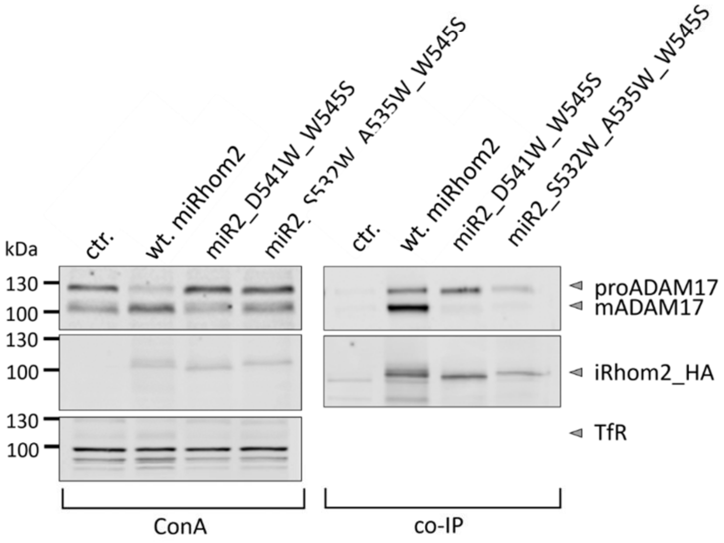

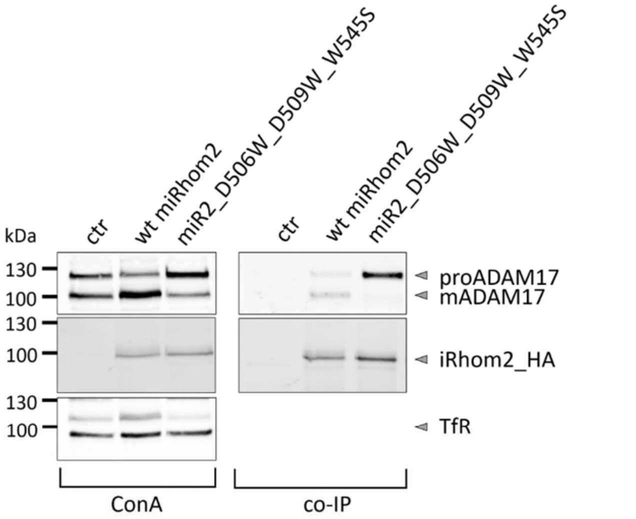

(B) **IRHD** of murine iRhom2: WPDDITKWPIC

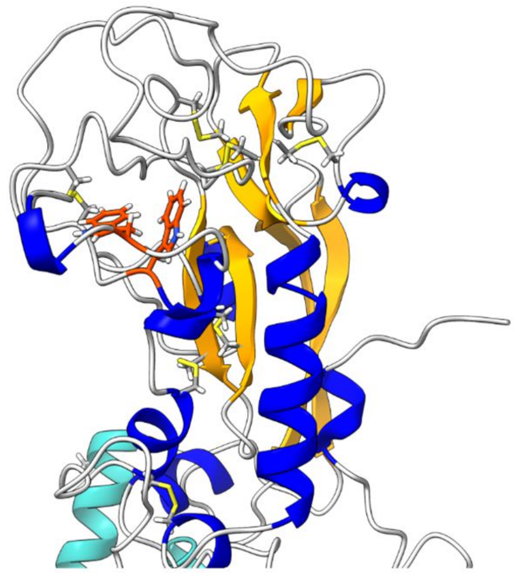

TSR1 of human UNC5 (4v2a): WSEWSPW

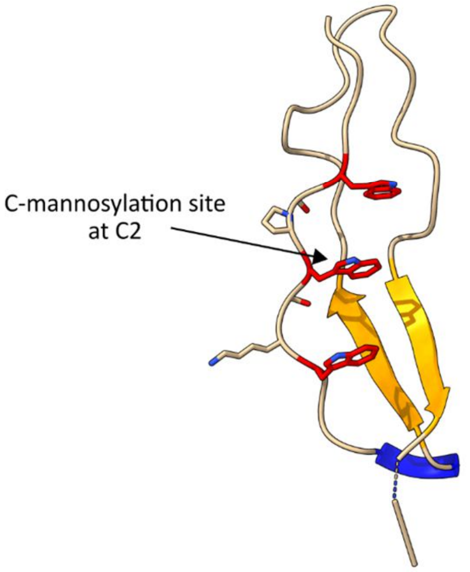

Figure S4: **(A)** Quantification of coIPs (Fig. 4A). **(B)** Quantification of coIPs (Fig. 4F). **(C)** Influence of miR2_hvGS and miR2_W538S on ADAM17-mediated shedding was analysed by performing an alkaline phosphatase (AP) assay in HEK293 cells. Cells expressing GFP were used as negative controls (ctr.). The ADAM17 substrate amphiregulin (AREG) tagged with AP was used. ADAM17 activity was additionally stimulated by TRAP6 or additionally inhibited by marimastat. Cells were incubated for 2 h under indicated treatment. n = 4. **(D)** Immunoblots to analyse the effects of wt murine iRhom2 (miR2), miR2_W538S or miR2_hvGS expression in MEFs derived from mice deficient in iRhom1 and iRhom2 on ADAM17 maturation. n = 3. **(E)** Influence of wt miR2, miR2_hvGS and miR2_W538S on ADAM17-mediated shedding was analysed by performing an alkaline phosphatase (AP) assay in MEFs derived from mice deficient in iRhom1 and iRhom2. Cells expressing GFP were used as negative controls (ctr.). The ADAM17 substrate TNFα tagged with AP was used. ADAM17 activity was additionally stimulated by the phorbol ester PMA or additionally inhibited by TAPI1. Cells were incubated for 2 h under indicated treatment. n = 4.

Fig. S4

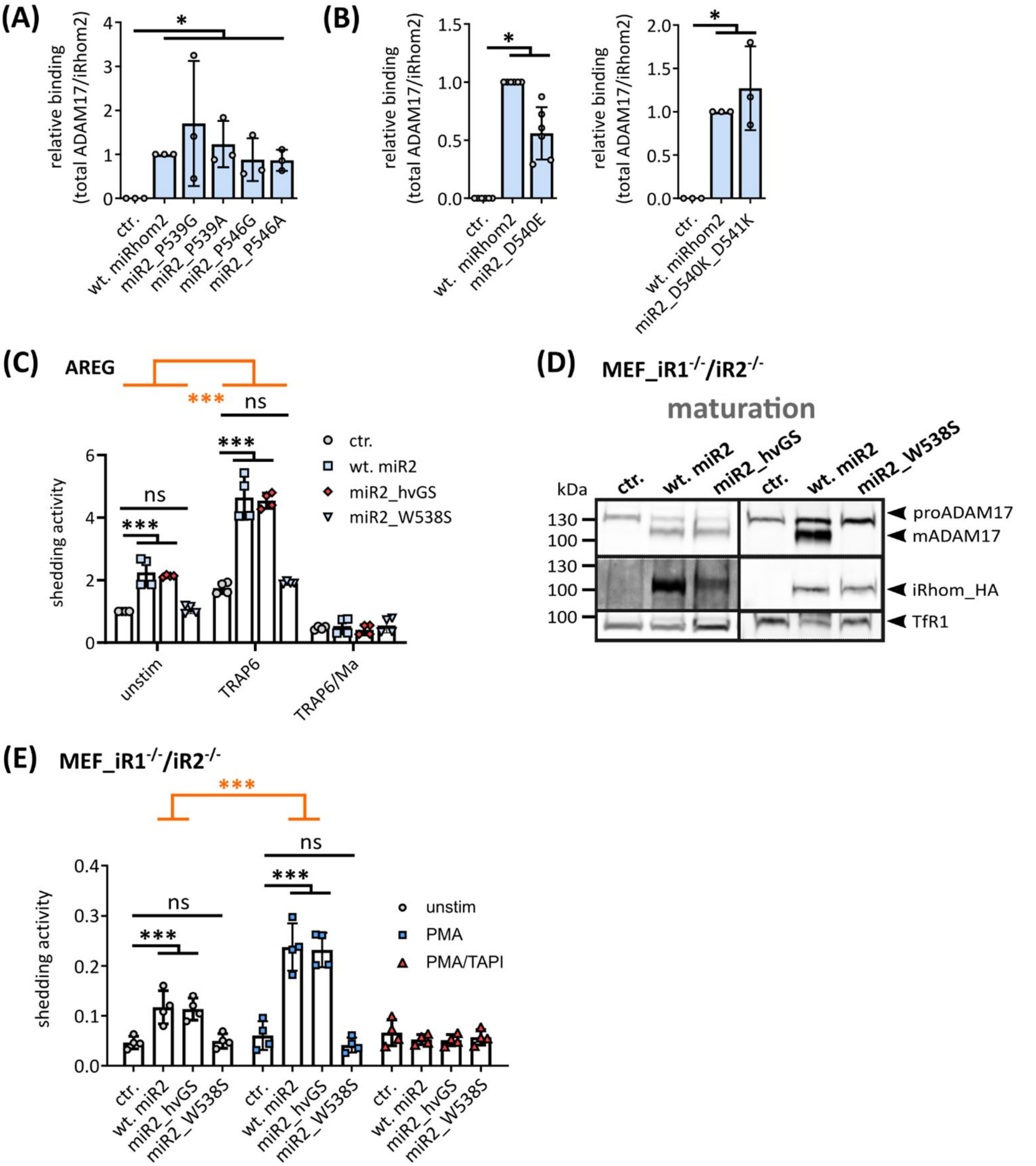

(F)

wt. miR2	**495**	FVKWQNDTGPS-DKSD----------------------------------LSQKQPSAVV
miR2_HyLmyc	**495**	FVKWQ**ATMEQKLISEEDLGTGSEQKLISEEDLGSAGEQKLISEEDLGGSGS**QKQPSAVV

Figure S5: (A) Cartoon representation of murine iRhom2 IRHD (salmon) and murine ADAM17 ectodomain (green) with and without ADAM17 prodomain (light green) in complex generated with AlphaFold Multimer. The predicted binding interfaces (hydrogen bonds only) of the relaxed complex structures are circled in red. A complete list of the putative interacting residue pairs between iRhom2 and ADAM17 can be found in Tab. S1. **(B)** The use of AlphaFold Multimer to model a complex consisting of murine iRhom2 and murine ADAM10 as a negative control resulted in no predicted interaction between both proteins, as evidenced by very high intermolecular PAE. **(C)** The *Drosophila* TNFα homologue Eiger is not a substrate of human ADAM17, as we have recently shown [97]. We have exchanged the TNF homology domain of Eiger for AP and inactivated two furin cleavage sites by mutation as described before [97]. By introducing an optimised ADAM17 cleavage site [98] at a distance from the membrane where the ADAM17 cleavage site in TNFα is located, Eiger is converted into a human ADAM17 substrate: A. Shifting the cleavage site away from the membrane increases the non-stimulated release of ADAM17: B to D. 10 µM TAPI1 was used as ADAM17 inhibitor. *Drosophila* Eiger constructs were used to generate the synthetic substrates to reduce interference from other factors in mammalian cells, and because of the very long flexible stalk region, which allowed the cleavage site to be shifted away from the membrane without risking steric hindrance of the domains.

Fig. S5

(A)

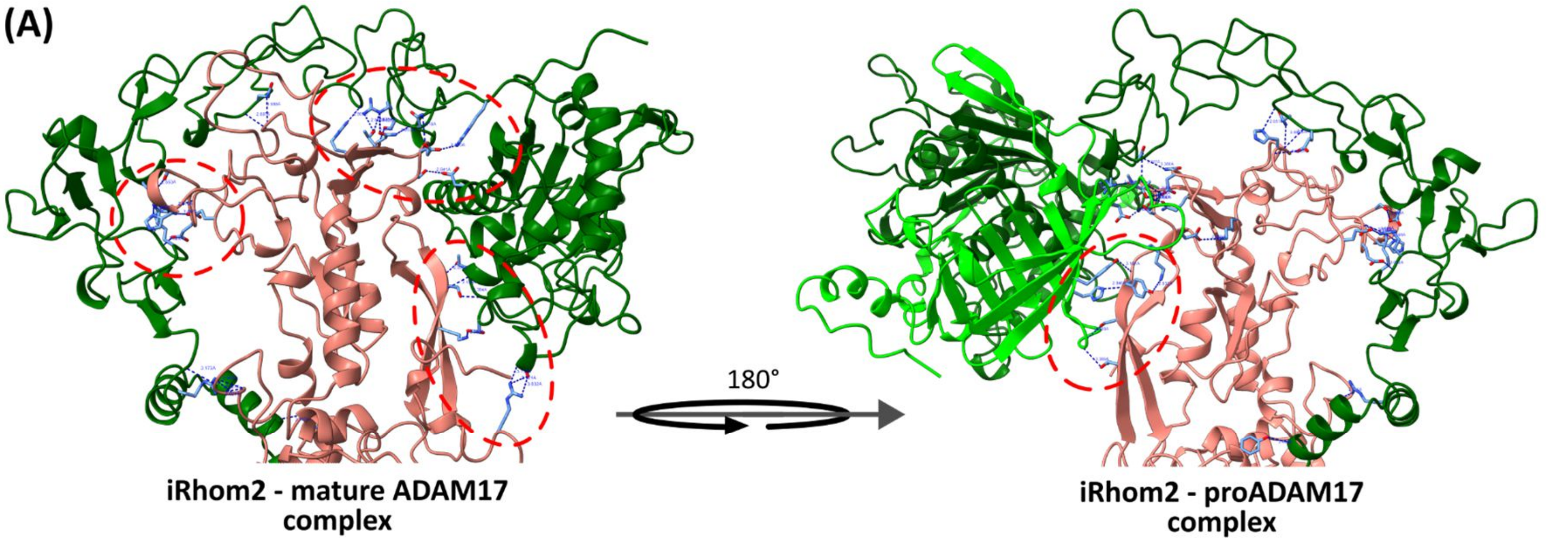

180°
iRhom2 - mature ADAM17 complex
iRhom2 - proADAM17 complex

(B)

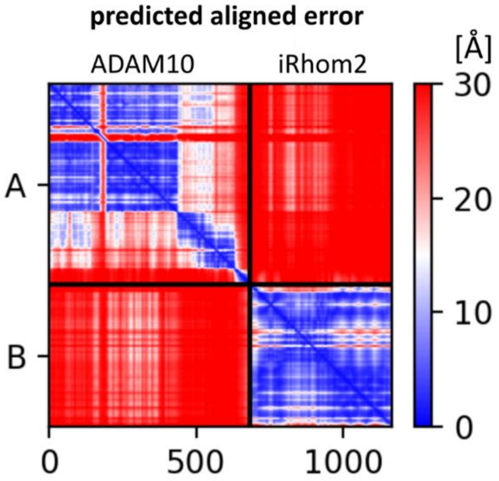

predicted aligned error
ADAM10
iRhom2
[Å]
30
20
10
0
A
B
0
500
1000

(C)

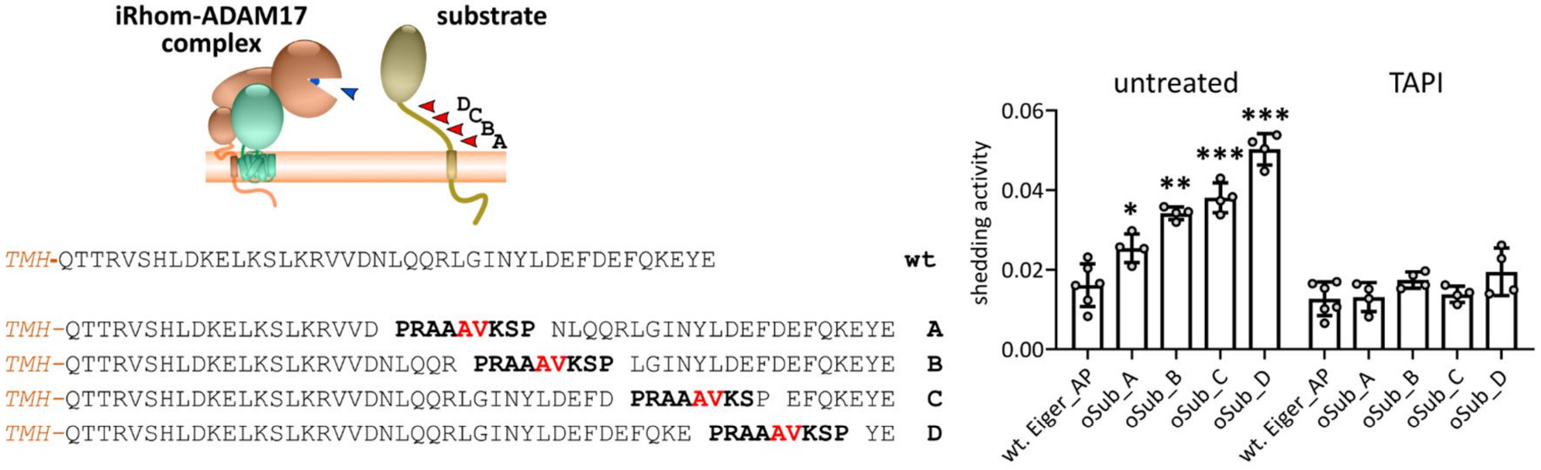

iRhom-ADAM17 complex
substrate
D
C
B
A
TMH-QTTRVSHLDKELKSLKRVVDNLQQRLGINYLDEFDEFQKEYE wt
TMH-QTTRVSHLDKELKSLKRVVD PRAAAVKSP NLQQRLGINYLDEFDEFQKEYE A
TMH-QTTRVSHLDKELKSLKRVVDNLQQR PRAAAVKSP LGINYLDEFDEFQKEYE B
TMH-QTTRVSHLDKELKSLKRVVDNLQQRLGINYLDEFD PRAAAVKSP EFQKEYE C
TMH-QTTRVSHLDKELKSLKRVVDNLQQRLGINYLDEFDEFQKE PRAAAVKSP YE D
untreated
TAPI
shedding activity
0.06
0.04
0.02
0.00
*
**
***
***
wt. Eiger_AP
oSub_A
oSub_B
oSub_C
oSub_D
wt. Eiger_AP
oSub_A
oSub_B
oSub_C
oSub_D

Figure S6: Overview of the substructures of the murine models generated with AlphaFold 2 and AlphaFold Multimer compared to experimentally solved structures from pdb [34].

Fig. S6

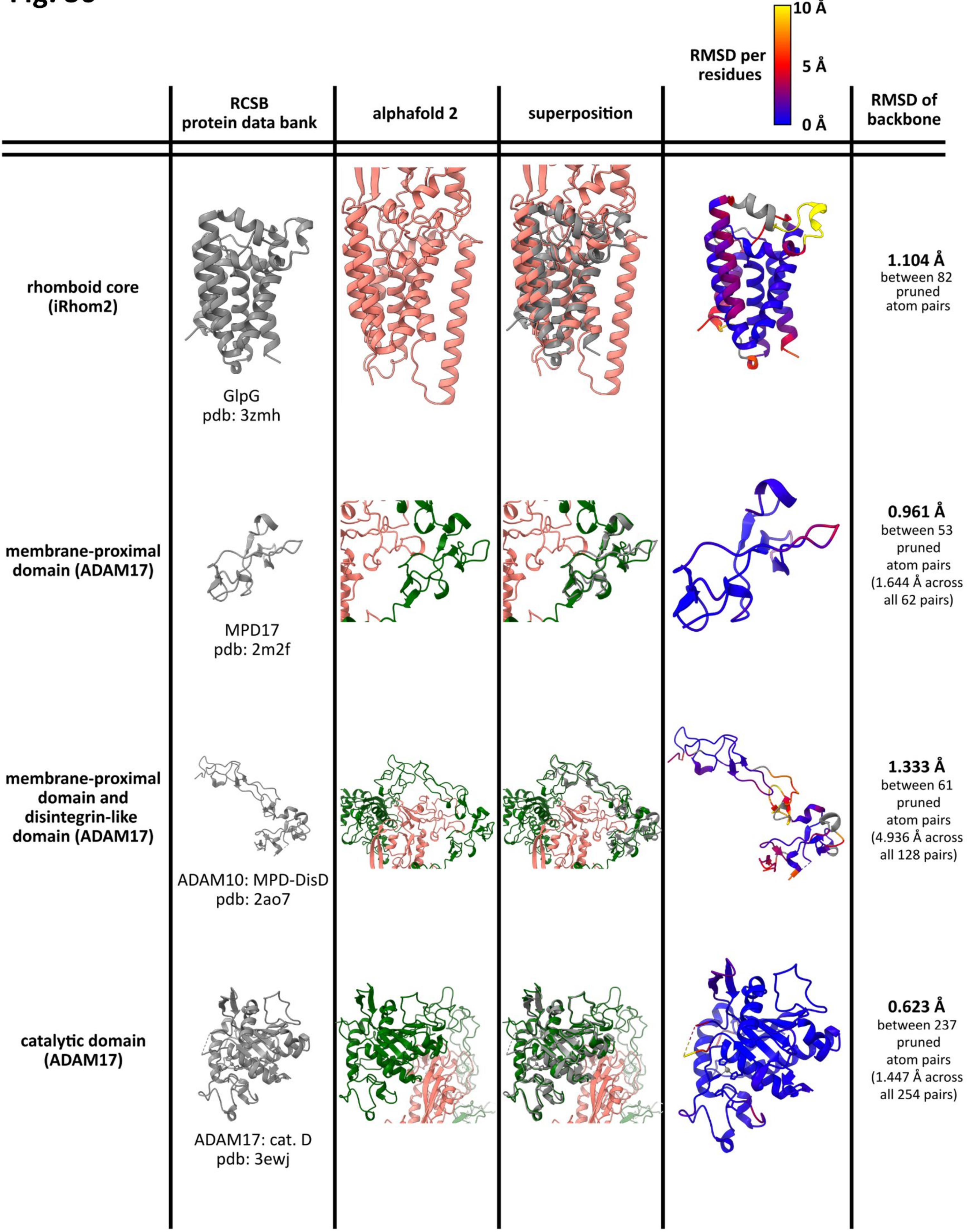
RMSD per residues
10 Å
5 Å
0 Å
RCSB protein data bank
alphafold 2
superposition
RMSD of backbone
rhomboid core (iRhom2)
GlpG
pdb: 3zmh
1.104 Å
between 82 pruned atom pairs
membrane-proximal domain (ADAM17)
MPD17
pdb: 2m2f
0.961 Å
between 53 pruned atom pairs (1.644 Å across all 62 pairs)
membrane-proximal domain and disintegrin-like domain (ADAM17)
ADAM10: MPD-DisD
pdb: 2ao7
1.333 Å
between 61 pruned atom pairs (4.936 Å across all 128 pairs)
catalytic domain (ADAM17)
ADAM17: cat. D
pdb: 3ewj
0.623 Å
between 237 pruned atom pairs (1.447 Å across all 254 pairs)

Figure S7: (A) PAE [Å] of the different predicted poses of the iRhom2-matureADAM17 complex predicted with AlphaFold Multimer v2. A: iRhom2, sequence: uniport entry Q80WQ6; B: ADAM17, sequence: uniport entry Q9Z0F8. **(B)** PAE [Å] of the different predicted poses of the iRhom2-matureADAM17 complex predicted with AlphaFold Multimer v3. A: iRhom2, B: ADAM17. **(C)** PAE [Å] of the different predicted poses of the iRhom2-proADAM17 complex predicted with AlphaFold Multimer v2. A: iRhom2, B: ADAM17. **(D)** Comparison/superposition of all ranked structure models/poses of the iRhom2-matureADAM17 complex and the iRhom2-proADAM17 complex predicted with AlphaFold Multimer v2. Structures are shown in cartoon representation.

Fig. S7

(A)

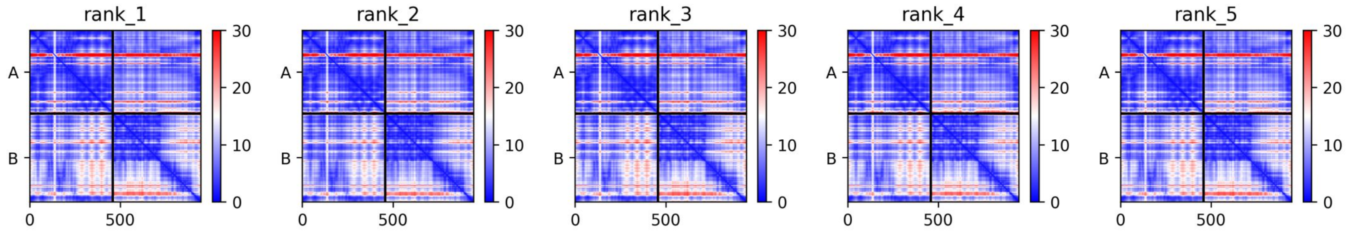
rank_1
rank_2
rank_3
rank_4
rank_5
A
B
0
500
30
20
10
0

(B)

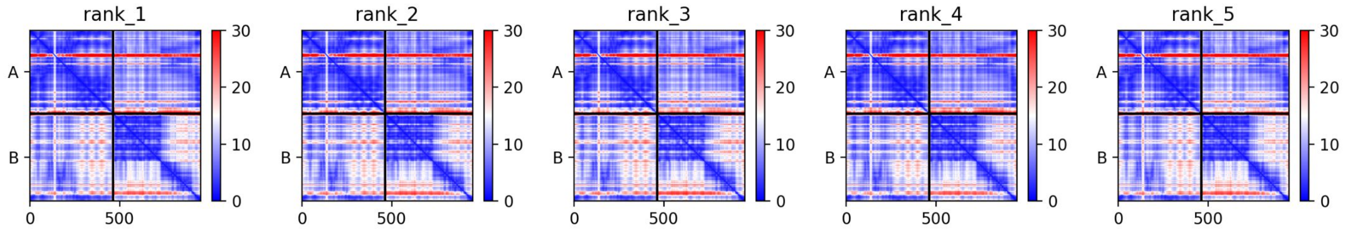
rank_1
rank_2
rank_3
rank_4
rank_5
A
B
0
500
30
20
10
0

(C)

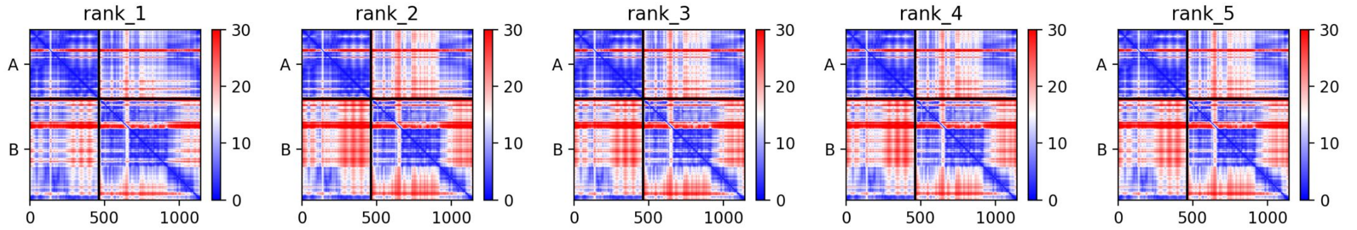
rank_1
rank_2
rank_3
rank_4
rank_5
A
B
0
500
1000
30
20
10
0

(D)

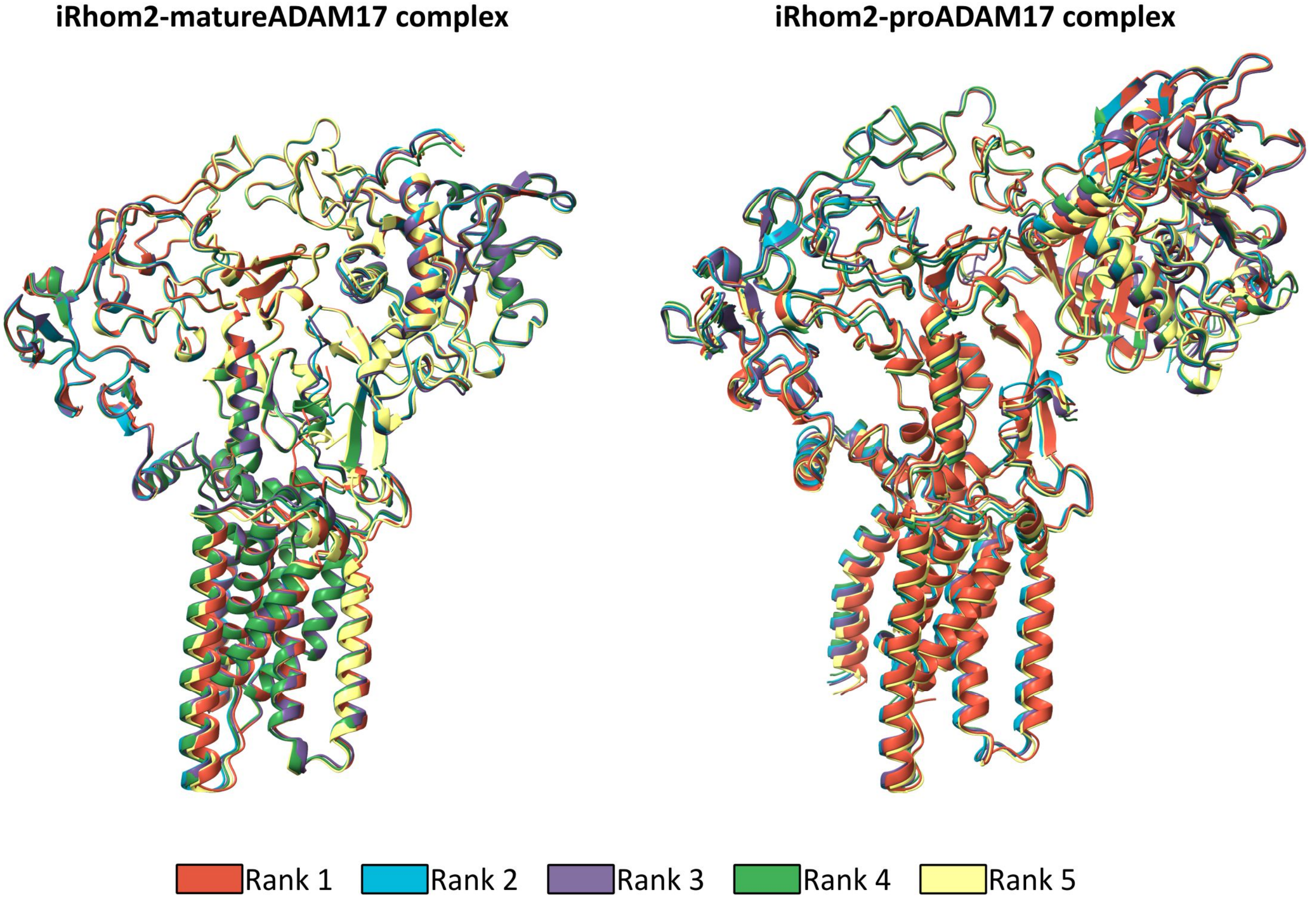
iRhom2-matureADAM17 complex
iRhom2-proADAM17 complex
Rank 1
Rank 2
Rank 3
Rank 4
Rank 5

Figure S8: Comparison/superposition and PAE [Å] of the different predicted poses of the indicated iRhom-matureADAM17 complexes predicted with AlphaFold Multimer v2. **(A)** human iRhom1 sequence: uniport entry Q96CC6; human ADAM17 sequence: uniport entry P78536. **(B)** human iRhom2 sequence: uniport entry Q6PJF5; human ADAM17 sequence: uniport entry P78536. **(C)** murine iRhom1 sequence: uniport entry Q6PIX5; murine ADAM17 sequence: uniport entry Q9Z0F8. **(D)** Caenorhabditis elegans iRhom sequence: uniport entry Q9U2S3; Caenorhabditis elegans ADAM17 sequence: uniport entry Q94316.

Fig. S8

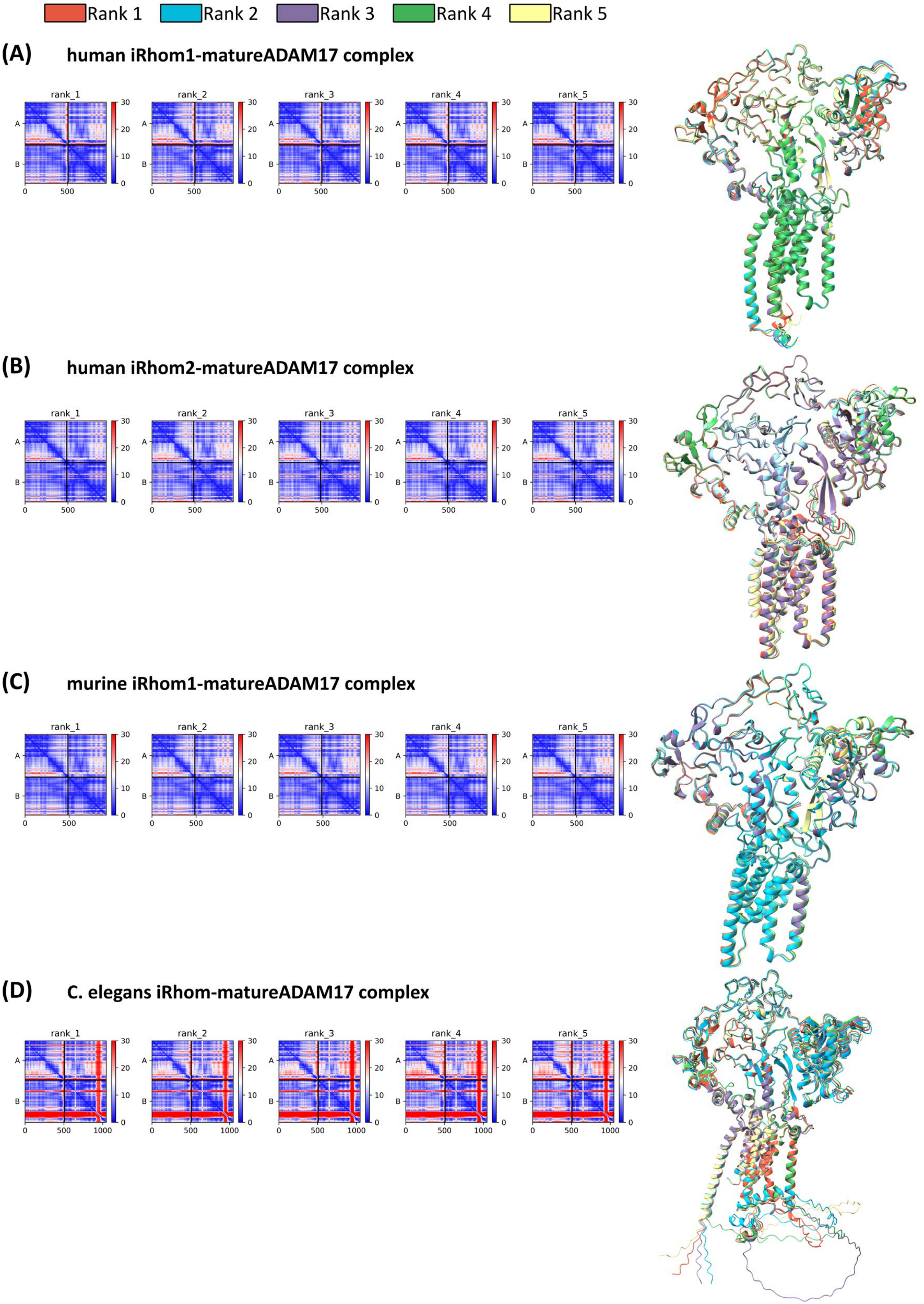
Rank 1
Rank 2
Rank 3
Rank 4
Rank 5
(A)
human iRhom1-matureADAM17 complex
rank_1
rank_2
rank_3
rank_4
rank_5
(B)
human iRhom2-matureADAM17 complex
rank_1
rank_2
rank_3
rank_4
rank_5
(C)
murine iRhom1-matureADAM17 complex
rank_1
rank_2
rank_3
rank_4
rank_5
(D)
C. elegans iRhom-matureADAM17 complex
rank_1
rank_2
rank_3
rank_4
rank_5

Figure S9: Comparison/superposition of the rank-1 predicted poses of the indicated iRhom-matureADAM17 complexes predicted with AlphaFold Multimer v2 (see Fig. S7 and Fig. S8).

Fig. S9

surface representation of murine ADAM17 (red) and murine iRhom2 (purple)

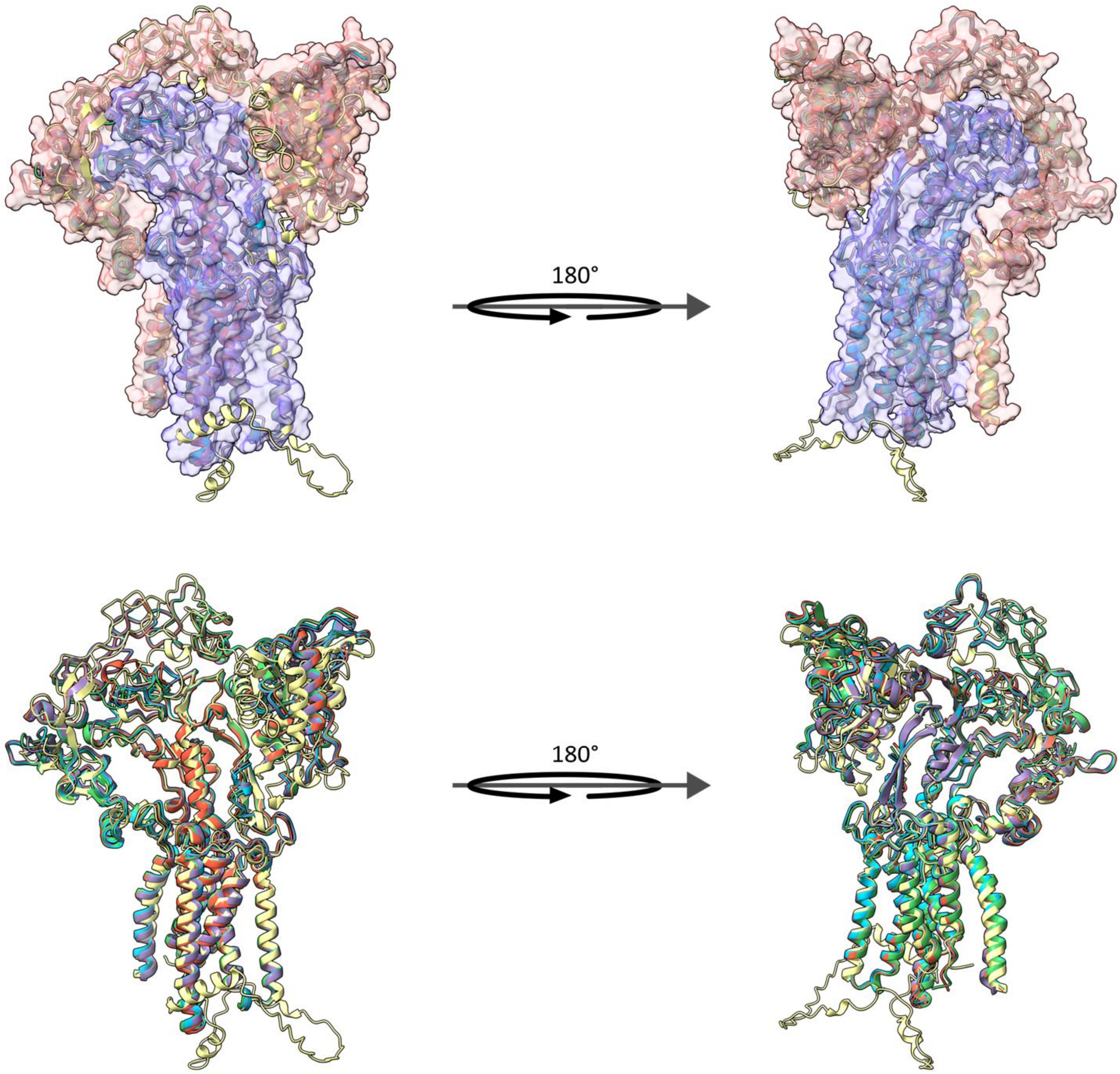

cartoon representation of iRhom-ADAM17 complex:

murine iRhom1-ADAM17 complex

murine iRhom2-ADAM17 complex

human iRhom1-ADAM17 complex

human iRhom2-ADAM17 complex

C. elegans iRhom-ADAM17 complex
