# Supplementary material for: A structural model of the iRhom–ADAM17 sheddase complex reveals functional insights into its trafficking and activity
Source: Cell Mol Life Sci. 2023 Apr 29;80(5):135. doi: 10.1007/s00018-023-04783-y (PMC10148629; doi:10.1007/s00018-023-04783-y)
Supplement: Supplementary file 12 — Supplementary file12 (PDF 246 KB) [file 18_2023_4783_MOESM12_ESM.pdf]

Table S1: Potential interaction sides between murine iRhom2 and murine ADAM17. The relaxed structures (best predicted model/rank 1) of the iRhom2-mature-ADAM17 complex and the iRhom2-proADAM17 complex were used to investigate interaction interfaces and identify putative residues involved. The analysis was performed with ChimeraX and InterProSurf. Residues that have putative intermolecular interactions (hydrogen bonds, van der Waal contacts) with each other are listed with their position in the respective substructure/domain. TMH = transmembrane helix; CANDIS = Conserved ADAM-SeventeeN Dynamic Interaction Sequence.

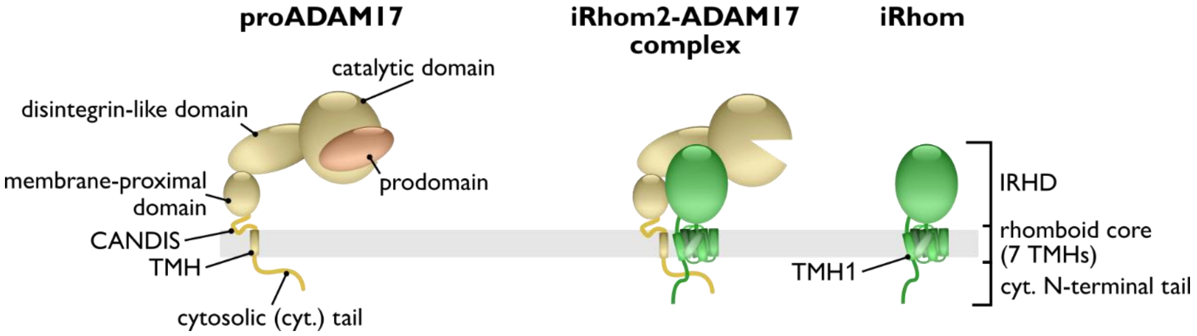

residues in murine iRhom2		residues in ADAM17	
**R373**	TMH1	D695	TMH
**Y375**	TMH1	V691	TMH
		W684	TMH
**Y378**	TMH1	W684	TMH
**W379**	TMH1	S680	TMH
		W684	TMH
		L681	TMH
**V383**	TMH1	L677	TMH
		S680	TMH
		V676	TMH
**I387**	TMH1	V673	CANDIS
		V676	TMH
**L390**	TMH1	V676	TMH
**T394**	TMH1	I672	CANDIS
**Y395**	TMH1	A669	CANDIS
		V676	TMH
		I672	CANDIS
**T408**	IRHD	I672	CANDIS
		G121	prodomain
**L410**	IRHD	R241	catalytic domain
		H118	prodomain

		Y82	prodomain
		V120	prodomain
**L412**	IRHD	K57	prodomain
**R415**	IRHD	E253	catalytic domain
	IRHD	R257	catalytic domain
**G416**	IRHD	K57	prodomain
**V417**	IRHD	Y250	catalytic domain
		N249	catalytic domain
		E253	catalytic domain
		Y82	prodomain
		T84	prodomain
		H65	prodomain
**Y418**	IRHD	R241	catalytic domain
	IRHD	Y250	catalytic domain
**Y418**	IRHD	Y82	prodomain
		H118	prodomain
		K57	prodomain
**E419**	IRHD	T396	catalytic domain
**S420**	IRHD	T396	catalytic domain
		P123	prodomain
**K422**	IRHD	D443	catalytic domain
		D124	prodomain
		P123	prodomain
**H440**	IRHD	D670	CANDIS
**L441**	IRHD	D670	CANDIS
		I672	CANDIS
**N475**	IRHD	N480	catalytic domain
		S481	disintegrin-like domain
		R482	disintegrin-like domain
**D476**	IRHD	S481	disintegrin-like domain
		S62	prodomain
		A63	prodomain
		T64	prodomain
**R477**	IRHD	P520	disintegrin-like domain
**S489**	IRHD	D256	catalytic domain

		E253	catalytic domain
		E88	prodomain
**E490**	IRHD	R473	catalytic domain
		N475	catalytic domain
		R482	disintegrin-like domain
**T491**	IRHD	N480	catalytic domain
		D256	catalytic domain
		I252	catalytic domain
		E88	prodomain
		N475	catalytic domain
**L492**	IRHD	I252	catalytic domain
		T64	prodomain
		S86	prodomain
**R525**	IRHD	A540	disintegrin-like domain
		T541	disintegrin-like domain
		L568	disintegrin-like domain
**T526**	IRHD	I538	disintegrin-like domain
		A540	disintegrin-like domain
**E528**	IRHD	R625	MPD
		L568	disintegrin-like domain
**E529**	IRHD	R625	MPD
		K626	MPD
**P530**	IRHD	G627	MPD
		K628	MPD
		K626	MPD
**A531**	IRHD	L568	disintegrin-like domain
		K628	MPD
		D569	disintegrin-like domain
**S533**	IRHD	D569	disintegrin-like domain
**A535**	IRHD	C591	MPD
		A592	MPD
**H536**	IRHD	K628	MPD
		P629	MPD
		L624	MPD
**E550**	IRHD	R625	MPD

**S554**	IRHD	E536	disintegrin-like domain
**H556**	IRHD	E536	disintegrin-like domain
		C534	disintegrin-like domain
		Q535	disintegrin-like domain
**T557**	IRHD	Q535	disintegrin-like domain
		I538	disintegrin-like domain
**K565**	IRHD	D59	prodomain
**L613**	IRHD	D670	CANDIS
		N671	CANDIS
**L614**	IRHD	R651	CANDIS
		F667	CANDIS
**P615**	IRHD	R651	CANDIS
**L617**	IRHD	Q646	CANDIS
		V645	MPD
		V648	CANDIS
		R651	CANDIS
**N618**	IRHD	V645	MPD
**P619**	IRHD	R644	MPD
**L630**	IRHD	R473	catalytic domain
		V673	CANDIS
**F633**	IRHD	V673	CANDIS
		L677	TMH
